# Identification of common cardiometabolic alterations and deregulated pathways in mouse and pig models of aging

**DOI:** 10.1111/acel.13203

**Published:** 2020-07-30

**Authors:** Víctor Fanjul, Inmaculada Jorge, Emilio Camafeita, Álvaro Macías, Cristina González‐Gómez, Ana Barettino, Beatriz Dorado, María Jesús Andrés‐Manzano, José Rivera‐Torres, Jesús Vázquez, Carlos López‐Otín, Vicente Andrés

**Affiliations:** ^1^ Centro Nacional de Investigaciones Cardiovasculares Carlos III (CNIC) Madrid Spain; ^2^ Departamento de Bioquímica y Biología Molecular Facultad de Medicina Instituto Universitario de Oncología Universidad de Oviedo Oviedo Spain; ^3^ Centro de Investigación Biomédica en Red Enfermedades Cardiovasculares (CIBERCV) Spain; ^4^ Centro de Investigación Biomédica en Red Enfermedades Cáncer (CIBERONC) Spain; ^5^ Present address: Facultad de Ciencias Biomédicas y de la Salud Universidad Europea de Madrid (UEM) Madrid Spain

**Keywords:** aging, cardiometabolic disease, HGPS, Hutchinson–Gilford progeria syndrome, mouse models, pathophysiology, pig models, proteomics

## Abstract

Aging is the main risk factor for cardiovascular and metabolic diseases, which have become a global concern as the world population ages. These diseases and the aging process are exacerbated in Hutchinson–Gilford progeria syndrome (HGPS or progeria). Here, we evaluated the cardiometabolic disease in animal models of premature and normal aging with the aim of identifying alterations that are shared or specific to each condition. Despite differences in body composition and metabolic markers, prematurely and normally aging mice developed heart failure and similar cardiac electrical abnormalities. High‐throughput proteomics of the hearts of progeric and normally aged mice revealed altered protein oxidation and glycation, as well as dysregulated pathways regulating energy metabolism, proteostasis, gene expression, and cardiac muscle contraction. These results were corroborated in the hearts of progeric pigs, underscoring the translational potential of our findings, which could help in the design of strategies to prevent or slow age‐related cardiometabolic disease.

## Graphical abstract

We have evaluated cardiac and metabolic disease in animal models of aging. Despite differences in body composition, progeric and normally aged mice developed heart failure and similar cardiac electrical alterations. High‐throughput proteomics of the hearts of these animals revealed dysregulation of energy metabolism, proteostasis, gene expression, and cardiac contraction pathways. These results were corroborated in progeric pigs, underscoring the translational potential of our findings.

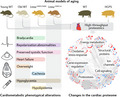

## 1 INTRODUCTION

Over the past two centuries, life expectancy has increased substantially, and this trend is projected to continue in most countries (Christensen, Doblhammer, Rau, & Vaupel, 2009; Kontis et al., 2017). Consequently, the world population is progressively aging. Aging leads to a decline in quality of life and poses one of the major socioeconomic challenges of our time. Aging is the main risk factor for cardiovascular disease (CVD), cancer, metabolic disorders, and neurodegeneration (Guzman‐Castillo et al., 2017; Kennedy et al., 2014). These diseases have therefore become a global concern, and among them, CVD is the leading cause of death in developed countries (Roth et al., 2017).

Current understanding of the molecular mechanisms underlying aging has in part been possible through research into Hutchinson–Gilford progeria syndrome (HGPS). This exceptionally rare (prevalence of 1 in 20 million), autosomal dominant disease is caused by a point mutation in exon 11 of the *LMNA* gene, which provokes the production of progerin, an aberrant form of lamin A. Progerin accumulation in the nuclear envelope causes structural defects, chromatin disorganization, and DNA damage (Dorado & Andrés, 2017; Gordon, Rothman, López‐Otín, & Misteli, 2014). Within the first 2 years of life, affected children begin to present symptoms such as failure to thrive and skin abnormalities. Patients progressively manifest other signs of premature aging, including alopecia, joint stiffness, osteopenia, and sarcopenia (Merideth et al., 2008). HGPS individuals also develop cardiometabolic disease, encompassing loss of subcutaneous fat, generalized arteriosclerosis, diastolic dysfunction, cardiac repolarization abnormalities, and valve disease, resulting in death mainly from heart failure, myocardial infarction, or stroke at an average age of 14.6 years (Gerhard‐Herman et al., 2012; Gordon, Shappell, et al., 2018; Merideth et al., 2008; Prakash et al., 2018; Rivera‐Torres et al., 2016).

The similarities between premature and normal aging extend to the molecular level, with all hallmarks of physiological aging also identified in HGPS (Carrero, Soria‐Valles, & López‐Otín, 2016; López‐Otín, Blasco, Partridge, Serrano, & Kroemer, 2013). Moreover, progerin is produced at low levels in normally aging individuals (McClintock et al., 2007; Rodriguez, Coppedè, Sagelius, & Eriksson, 2009; Scaffidi, 2006). Nevertheless, some key aspects of aging are not recapitulated in HGPS. Most prominent among these are the absence in HGPS of neurodegeneration and immune system deficits, and the low frequency of cancer (Gordon et al., 2014). Furthermore, HGPS patients are not typically exposed in the long term to the unhealthy behaviors and environmental factors that promote age‐related diseases (inadequate nutrition, sedentary lifestyle, stress, smoking, etc.) (Fontana, 2018). However, these dissimilarities could prove advantageous in aging research because they allow progeria to be used to study age‐related cardiometabolic disease without interference from certain comorbidities and environmental risk factors.

There are only about 150 identified children living with HGPS worldwide (progeriaresearch.org). The development of appropriate models of the disease has therefore been a critical step toward understanding its etiology and developing therapeutic strategies. Among the available mouse models of HGPS, the progerin‐expressing *Lmna^G609G^* knock‐in strain recapitulates most of the clinical manifestations of this rare disease, such as growth impairment, bone abnormalities, lipodystrophy, CVD, and premature death (Osorio et al., 2011). Recent studies have focused on identifying cardiac, arterial, and metabolic alterations in this model (Bárcena et al., 2018; Del Campo et al., 2019; Hamczyk et al., 2018, 2019; Osorio et al., 2011). However, much remains unknown about the mechanisms underlying these abnormalities and their connection to normal aging, particularly in the heart. Here, we performed a comprehensive cardiac and metabolic characterization of *Lmna^G609G^* mice with young and old wild‐type (WT) mice. To investigate protein alterations associated with cardiometabolic disease during aging, we performed high‐throughput proteomics to identify molecular changes in the mouse heart that are common or specific to premature and normal aging. We also analyzed the cardiac proteome in a minipig model of HGPS (Dorado et al., 2019) to identify conserved alterations that might have high translational value for the development of rejuvenation and prevention strategies.

## 2 RESULTS

### 2.1 Progeric and normally aged mice develop heart failure and similar cardiac electrical alterations

The median survival of wild‐type (WT) mice is 28 months (Flurkey, Currer, & Harrison, 2007). We therefore analyzed 20‐month‐old WT mice (“old WT”) as a model of normal aging. To model HGPS and assess progerin dose‐dependent effects, we used 4‐month‐old heterozygous *Lmna^G609G^* ^/^ *^wt^* and homozygous *Lmna^G609G^* ^/^ *^G609G^* mice (lifespan ~12 and ~5 months, respectively). Four‐month‐old WT animals (“young WT”) were used as controls.

**FIGURE 1:**
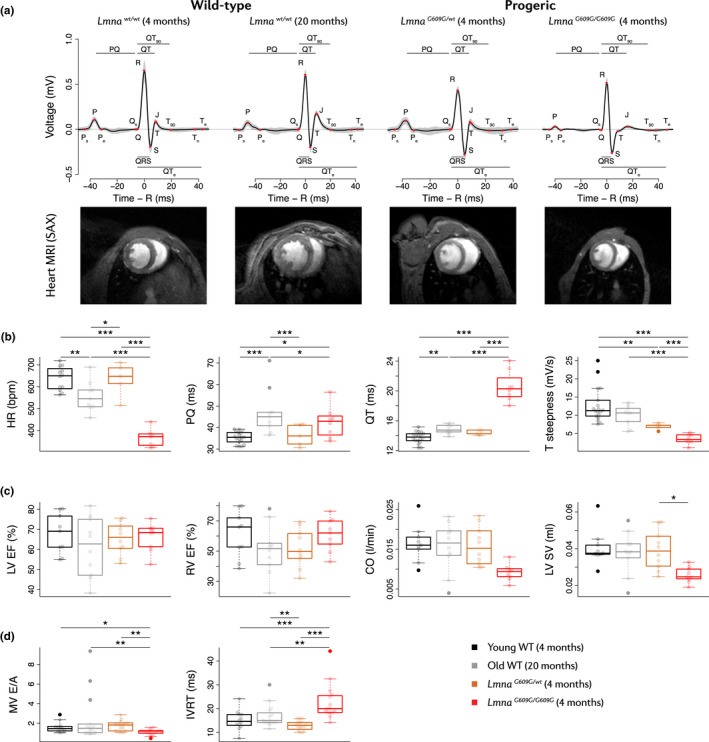
Bradycardia, HFpEF, and repolarization abnormalities in progeric and old WT mice. Studies were carried out in 4‐month‐old progeric *Lmna^G609G^* ^/^ *^G609G^* and *Lmna^G609G^* ^/^ *^wt^* mice and in 4‐month‐old (Young) and 20‐month‐old (Old) WT mice. (a) Representative ECGs and MRI scans showing heart ventricles in short‐axis (SAX) view (blood in white). (b) HR and ECG parameters (*n* = 8 *Lmna^G609G^* ^/^ *^G609G^*, 5 *Lmna^G609G^* ^/^ *^wt^*, 18 young WT, and 9 Old WT mice). Measurements were performed at least 3 times within a month. (c) Cardiac function parameters derived from MRI (*n* = 10 mice per group). Body weight was considered as a covariate in the statistical analysis of CO and SV. (d) Echocardiographic parameters (*n* = 12–16 mice per group). *p < 0.05; **p < 0.01; ***p < 0.001.

To tackle age‐related cardiac and metabolic alterations in these models, we first performed electrocardiography (ECG) experiments and assessed heart rate (HR). Old WT and *Lmna^G609G^* ^/^ *^G609G^* mice both had a lower HR and longer PQ, QT, QT_90,_ and QT_e_ intervals than *Lmna^G609G^* ^/^ *^wt^* and young WT animals (Figure 1a,b, Figure S1A). ECG alterations were more severe in *Lmna^G609G^* ^/^ *^G609G^* mice, which also showed T‐wave flattening and retardation, manifested as a gap between the J and T waves (Figure 1b, Figure S1A). These observations are consistent with bradycardia and electrical conduction and repolarization abnormalities, previously described in HGPS patients and progeric *Zmpste24*‐deficient mice (Rivera‐Torres et al., 2016). Four‐month‐old *Lmna^G609G^* ^/^ *^wt^* animals had normal ECGs except for a mild but significant reduction in T‐wave steepness and a longer QT_90_ compared with young WT controls (Figure 1b, Figure S1A). QRS complex duration was normal in all groups (Figure S1A).

We next assessed cardiac function by magnetic resonance imaging (MRI). Systolic function was preserved in all the compared groups, indicated by normal values for both left ventricle (LV) and right ventricle (RV) ejection fractions (EFs) (Figure 1a,c). Differences in cardiac output (CO) and stroke volume (SV) were not significant when body weight was considered as a covariate in the statistical model (Figure 1c). Old WT mice also had a higher Fulton index and a higher RV‐to‐LV end‐diastolic volume (EDV) ratio than young WT mice, as well as a modest non‐significant reduction in RV EF, evidencing the enlarged RV of old WT mice (Figure 1c, Figure S1B). Further assessment of cardiac diastolic function by echocardiography revealed a reduced mitral valve (MV) E/A ratio and increased isovolumetric relaxation time (IVRT) in *Lmna^G609G^* ^/^ *^G609G^* mice (Figure 1d). Old WT mice showed a modest increase in IVRT, and although the E/A ratio appeared normal in most individuals, 3 out of 16 old WT mice presented restrictive LV filling (E/A > 3). These alterations are consistent with diastolic dysfunction.

To better understand the cardiac alterations of heterozygous progeric mice, we also performed and electro‐ and echocardiographic characterization of 10‐month‐old *Lmna^G609G^* ^/^ *^wt^* animals (“old *Lmna^G609G^* ^/^ *^wt^*”) (Figure S2). Old *Lmna^G609G^* ^/^ *^wt^* mice presented lower HR, longer QT, QT_90, _and QT_e_ intervals, and greater T‐wave flattening than age‐matched WT animals (Figure S2A). Moreover, old *Lmna^G609G^* ^/^ *^wt^* mice showed preserved EF, CO, and LV mass, but altered MV E/A and increased IVRT compared with WT controls (Figure S2B). These findings indicate that *Lmna^G609G^* ^/^ *^wt^* mice develop a late‐onset cardiac pathology similar to that of 4‐month‐old *Lmna^G609G^* ^/^ *^G609G^* mice.

Taken together, these results suggest that both progeric and normally aged mice develop heart failure with preserved ejection fraction (HFpEF), a typical condition in the elderly (Wong et al., 2016) and one of the main causes of death in HGPS (Gordon, Shappell, et al., 2018).

### 2.2 Progeric and normally aged mice present distinct metabolic abnormalities

**FIGURE 2:**
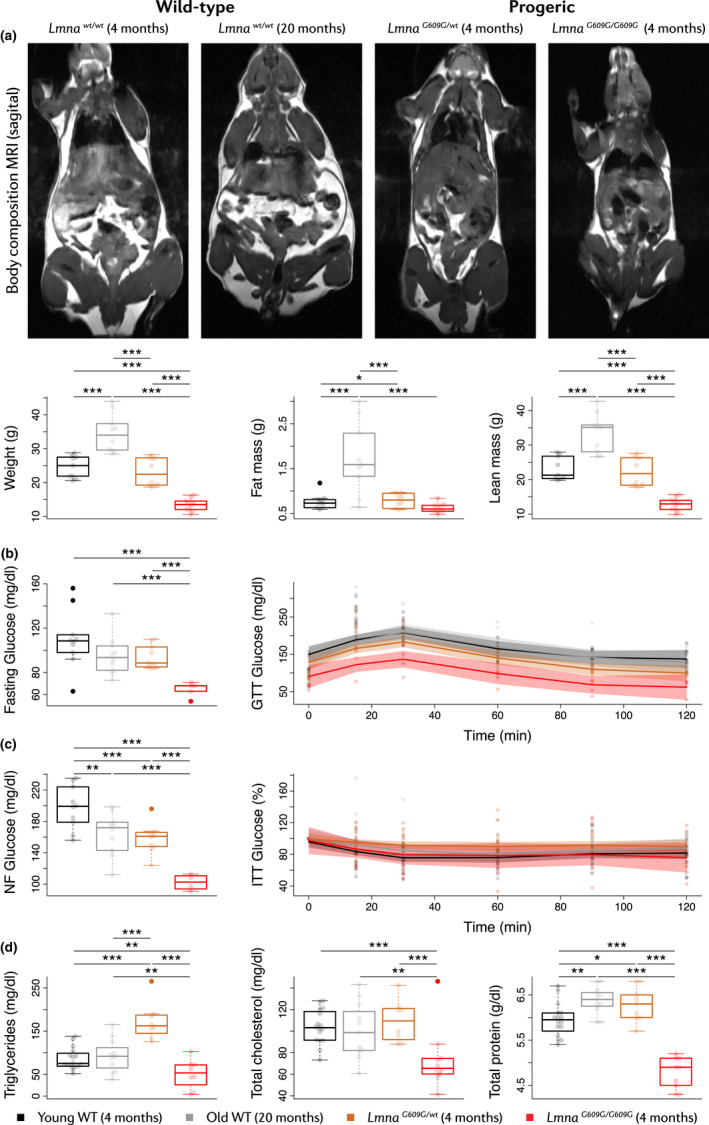
Systemic metabolic alterations in progeric and old WT mice. Studies were carried out in 4‐month‐old progeric *Lmna^G609G^* ^/^ *^G609G^* and *Lmna^G609G^* ^/^ *^wt^* mice and in 4‐month‐old (Young) and 20‐month‐old (Old) WT mice. (a) Whole‐body MRI (*n* = 10 mice per group). Images show representative examples of sagittal whole‐body cross‐sections (fat in white) and analysis of body composition. (b) GTT (with overnight fasting) and (c) ITT (non‐fasting: NF) (*n* = 5 or 10 mice per group). Glucose levels are presented at time 0 (left) and over the course of the experiment (right). (d) Biochemical analysis of metabolism‐related serum parameters (*n* = 10–16 mice per group). *p < 0.05; **p < 0.01; ***p < 0.001.

We next examined body composition by MRI and measured metabolic parameters in the normal and premature aging models. Old WT and young *Lmna^G609G^* ^/^ *^wt^* mice presented increased adiposity in relation to young WT mice (Figure 2a). The former also had higher body weight and lean mass than the other models (Figure 2a). In contrast, *Lmna^G609G^* ^/^ *^G609G^* animals were smaller and leaner than the other groups (Figure 2a). This indicates that whereas WT mice become obese during normal aging, progeric mice develop cachexia, like HGPS patients (Hennekam, 2006; Merideth et al., 2008).

Metabolic tests in *Lmna^G609G^* ^/^ *^G609G^* mice revealed basal hypoglycemia in both fasting and non‐fasting conditions and increased glucose tolerance during an intraperitoneal glucose tolerance test (GTT) (Figure 2b,c). Young *Lmna^G609G^* ^/^ *^wt^* and old WT animals also had lower basal glucose levels in non‐fasting conditions than young WT mice, and young *Lmna^G609G^* ^/^ *^wt^* mice had higher glucose tolerance in the GTT (Figure 2b,c). None of the groups showed insulin intolerance during an insulin tolerance test (ITT) (Figure 2c).

Biochemical analysis revealed extensive alterations in the serum of *Lmna^G609G^* ^/^ *^G609G^* mice, with low levels of triglycerides, total cholesterol, total protein, and albumin, as well as elevated levels of ureic nitrogen, transaminases, and creatine kinase (Figure 2d, Figure S3). Bilirubin and alkaline phosphatase levels appeared normal, therefore suggesting that these abnormalities are related not to liver damage but to cachexia (Figure S3). Old WT mice also had elevated serum transaminases levels (Figure S3); moreover, triglyceride and protein levels in young *Lmna^G609G^* ^/^ *^wt^* mice were significantly higher than in young WT animals (Figure 2d).

Metabolic characterization also revealed reduced body weight and lower serum levels of glucose and total cholesterol in 10‐month‐old *Lmna^G609G^* ^/^ *^wt^* mice compared with age‐matched WT controls (Figure S4). Old *Lmna^G609G^* ^/^ *^wt^* mice also showed a tendency toward reduced serum levels of proteins, and increased levels of ureic nitrogen and transaminases (Figure S4B). These findings suggest heterozygous progeric mice develop late‐onset metabolic alterations similar to those of *Lmna^G609G^* ^/^ *^G609G^* mice.

Collectively, our results show that both progeric and old WT mice develop conspicuous cardiac and metabolic disorders characterized by bradycardia, cardiac repolarization abnormalities, HFpEF, hypoglycemia, and altered body composition. Furthermore, whereas old WT mice become overweight and display an enlarged RV, young *Lmna^G609G^* ^/^ *^wt^* mice show very mild alterations, and *Lmna^G609G^* ^/^ *^G609G^* and old *Lmna^G609G^* ^/^ *^wt^* animals develop hypolipidemia and cachexia.

### 2.3 Quantitative proteomics reveals conspicuous alterations in the progeric and normally aged mouse heart

**FIGURE 3:**
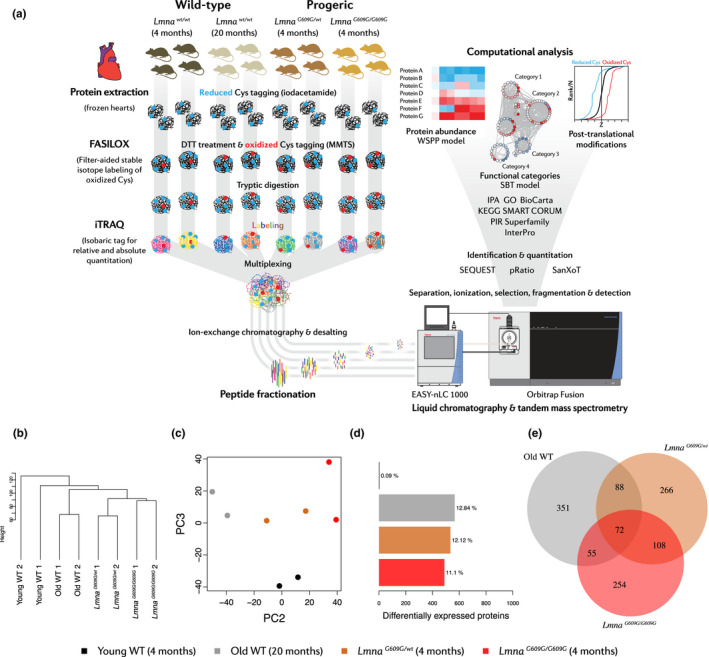
Cardiac high‐throughput proteomics in progeric and old WT mice. Studies were carried out in 4‐month‐old progeric *Lmna^G609G^* ^/^ *^G609G^* and *Lmna^G609G^* ^/^ *^wt^* mice and in 4‐month‐old (Young) and 20‐month‐old (Old) WT mice. (a) Experimental design of high‐throughput quantitative proteomic analysis based on the FASILOX method and iTRAQ‐multiplexed labeling. Two samples were analyzed for each experimental condition, each one consisting of a pool of 2 hearts. (b) Hierarchical clustering and (c) PCA of the samples (filtering for proteins with >1 unique peptide identified). (d) Bar plots representing the number of differentially expressed proteins in each condition. (e) Venn diagram showing common dysregulated proteins in each condition. A protein was considered significantly changed when it was quantified with >1 unique peptide and the |mean Zq| between replicates was >1.5.

To identify protein alterations associated with cardiometabolic disease in progeria and aging, we carried out high‐throughput, quantitative proteomics on heart tissue from 4‐month‐old progeric *Lmna^G609G^* ^/^ *^wt^* and *Lmna^G609G^* ^/^ *^G609G^* mice and age‐matched and 20‐month‐old WT animals (Figure 3a). We used male mice, with two samples for each condition, each sample consisting of a pool of 2 hearts (*n* = 4 mice per group). Mass spectrometry using multiplexed isobaric labeling followed by peptide fractionation detected and quantified 7102 proteins with a FDR<1%; of these proteins, 4477 were identified with more than one unique peptide (Table S1).

Despite the expected basal variability, hierarchical clustering and principal component analysis (PCA) showed a clear separation among the four experimental groups, highlighting the existence of proteome signatures specific to each condition (Figure 3b,c). Furthermore, these analyses revealed that the heart proteome of old WT mice is more similar to that of progeric mice than to that of young WT animals and that the proteomic profile of *Lmna^G609G^* ^/^ *^G609G^* animals most closely resembles that of young *Lmna^G609G^* ^/^ *^wt^* mice (Figure 3b,c). Confirming these findings, the protein abundance changes in biological replicates from the same condition showed a clearly higher correlation (*R* ^2^ > 0.73) than abundance changes between different conditions (*R* ^2^ < 0.54) (Figure S5).

Taking young WT mice as a reference, the number of altered proteins was found to be similar across the different comparisons (11%–13% of the total proteome, only considering proteins identified with >1 unique peptide and with a mean absolute Z score |mean Zq| higher than 1.5) (Figure 3d). In total, 72 proteins were commonly dysregulated in all three models, 180 in both progeric mouse strains, 127 in normally aged versus *Lmna^G609G^* ^/^ *^G609G^* animals, and 160 in normally aged versus young *Lmna^G609G^* ^/^ *^wt^* animals (Figure 3e). A curated list of differentially abundant proteins is provided as a heat map in Figure S6.

### 2.4 Proteomic changes in the progeric and normally aged mouse heart are related to cardiometabolic disease

**FIGURE 4:**
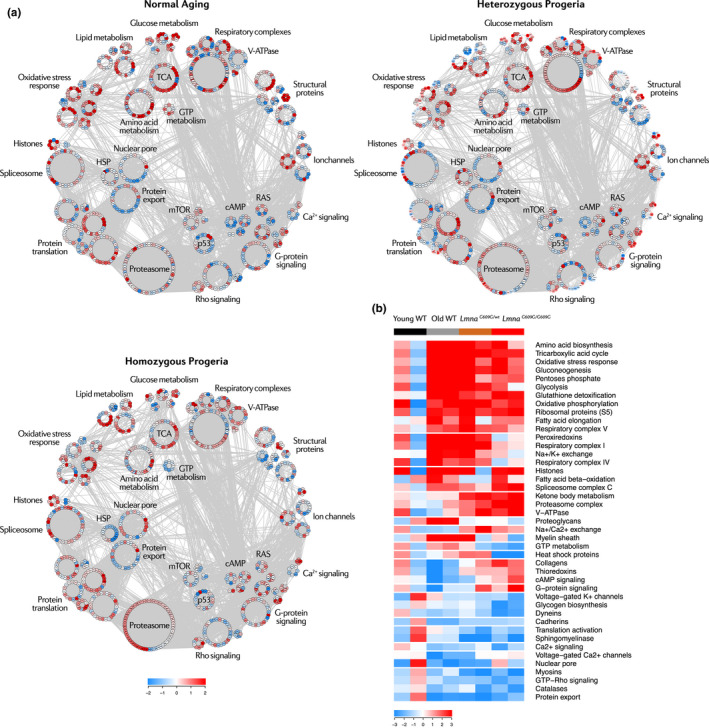
Systems biology overview of the aging mouse heart proteome. Studies were carried out in 4‐month‐old progeric *Lmna^G609G^* ^/^ *^G609G^* and *Lmna^G609G^* ^/^ *^wt^* mice and in 4‐month‐old (Young) and 20‐month‐old (Old) WT mice. (a) Proteomic landscape showing the main categories altered in the progeric and old WT mouse heart. Each dot represents a protein, color‐coded according to mean relative abundance in the aging proteome. Color intensity is maximal when |mean Zq| ≥ 2. HSP, heat shock proteins; TCA, tricarboxylic acid. (b) Heatmap of curated categories with altered regulation in premature and/or normal aging models. Selected categories have |Zc| > 1.5 in both replicates or |mean Zc| > 1.5. Color intensity is maximal when |Zc| ≥ 3.

Systems biology analysis of the heart proteome using the Systems Biology Triangle (SBT) model (García‐Marqués et al., 2016; Trevisan‐Herraz et al., 2019) revealed the existence of coordinated age‐dependent protein changes in pathways related to energy metabolism, muscle contraction (cell structure, ion channels, and signaling), gene expression, proteostasis, and response to oxidative stress (Figure 4). Energy metabolism alterations were common to old WT mice young *Lmna^G609G^* ^/^ *^wt^* mice and to a lesser extent *Lmna^G609G^* ^/^ *^G609G^* mice (Figure 4, Figure S7). These alterations involved increased relative abundance of proteins related to glycolysis, gluconeogenesis, pentose phosphate synthesis, fatty acid metabolism, amino acid biosynthesis, tricarboxylic acid cycle, and oxidative phosphorylation. *Lmna^G609G^* ^/^ *^G609G^* mice were distinguished by lower relative abundance of glycogen biosynthesis proteins. Other metabolic abnormalities in both *Lmna^G609G^* models included increased relative abundance of ketone body metabolism proteins and vacuolar‐type H^+^‐ATPases (V‐ATPases) and decreased relative abundance of GTP and sphingomyelin metabolism proteins (Figure 4, Figure S7).

The aging heart also showed alterations in motor and structural proteins (Figure 4, Figure S8A). Myosins, dyneins, and cadherins showed decreased relative expression—the first two particularly in progeric mice and the third in old WT animals. Collagens were upregulated in the progeric heart, whereas some collagens were downregulated in the normally aged heart. Lastly, proteoglycans and myelins showed an increased relative abundance in old WT hearts. Ion channels and signaling pathways related to cardiac contraction, such as calcium, potassium, and Rho signaling, were downregulated in all aging groups, whereas Na^+^/K^+^ and Na^+^/Ca^2+^ exchangers were upregulated particularly in young *Lmna^G609G^* ^/^ *^wt^* mice (Figure 4, Figure S8B,C). Interestingly, G‐protein and cAMP signaling proteins were dysregulated in opposite directions in progeric and old WT mice. These results evidence that the heart undergoes remodeling in both premature and aging models through shared and distinct alterations in structural and motor proteins. Cardiac remodeling at the molecular level may be associated with the phenotype of HFpEF revealed by our *in vivo* experiments (Figure 1).

Analysis of gene expression regulation during aging revealed elevated abundance of spliceosome components and histones in old WT and progeroid mice (Figure 4, Figure S9A). All three models showed relative reductions in protein export and translation activation, and the *Lmna^G609G^* ^/^ *^G609G^* model showed low levels of heat shock proteins (HSP). Progeric and normally aged animals both showed increased relative abundance in proteasome complex and S5 ribosomal components (Figure 4, Figure S9A). Compared with young WT mice, the glutathione and peroxiredoxin systems were upregulated in the three aging groups (Figure 4, Figure S9B). Despite a downregulation of catalases, these results suggest that a compensatory antioxidant response is activated during both premature and normal aging.

### 2.5 Premature and normal aging are associated with protein oxidation and glycation changes in the mouse heart

**FIGURE 5:**
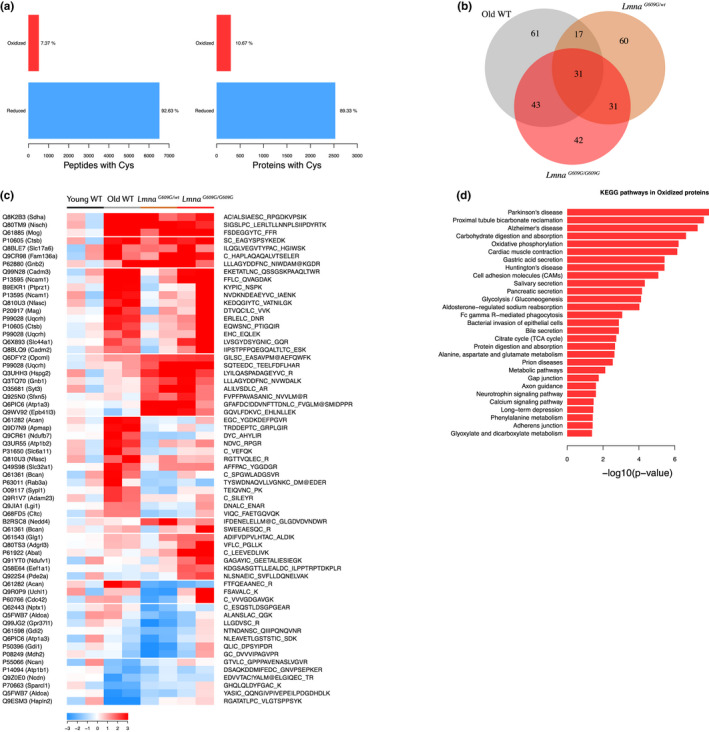
Abnormal protein oxidation in the aging mouse heart proteome. Studies were carried out in 4‐month‐old progeric *Lmna^G609G^* ^/^ *^G609G^* and *Lmna^G609G^* ^/^ *^wt^* mice and in 4‐month‐old (Young) and 20‐month‐old (Old) WT mice. (a) Bar plots representing number of peptides and proteins with oxidized or reduced Cys residues. (b) Venn diagram showing common dysregulated oxidized peptides in each condition. (c) Heatmap of curated oxidized peptides with altered expression. Color intensity is maximal when |Zpq| ≥ 3. (d) Gene enrichment analysis of proteins with altered oxidized peptides. A peptide was considered significantly changed when mean Zpq between replicates was >1.5. C_, oxidized Cys; C!, reduced Cys; M@, oxidized methionine.

We next assessed reversible protein cysteine oxidation and glycation in the aging heart by measuring alterations in abundance of posttranslationally modified peptides in relation to the levels of the corresponding proteins (Zpq) (Bagwan et al., 2018). Around 7% of the peptides bearing Cys residues were oxidized, affecting 11% of the proteins (Figure 5a). We found 31 peptides commonly dysregulated in the three aging conditions (|mean Zpq| > 1.5), 62 common to the two progeric groups, 74 common to normally aged and *Lmna^G609G^* ^/^ *^G609G^* mice, and 48 common to normally aged and young *Lmna^G609G^* ^/^ *^wt^* animals (Figure 5b). Enrichment analysis using the KEGG database revealed that in both premature and normal aging, the oxidized peptides with altered abundance belong mainly to mitochondrial and structural proteins involved in energy metabolism and cardiac muscle contraction (Figure 5c,d).

**FIGURE 6:**
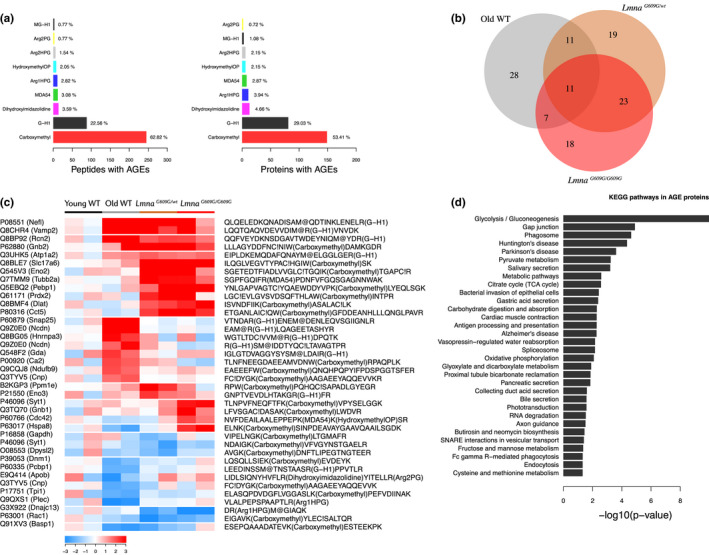
Altered protein glycation in the aging mouse heart proteome. Studies were carried out in 4‐month‐old progeric *Lmna^G609G^* ^/^ *^G609G^* and *Lmna^G609G^* ^/^ *^wt^* mice and in 4‐month‐old (Young) and 20‐month‐old (Old) WT mice. (a) Bar plots representing number of peptides and proteins with AGEs. (b) Venn diagram showing common dysregulated glycated peptides in each condition. (c) Heatmap of curated glycated peptides with altered expression. Color intensity is maximal when |Zpq| ≥ 3. (d) Gene enrichment analysis of proteins with altered glycated peptides. A peptide was considered significantly changed when mean Zpq between replicates was >1.5. C_, oxidized Cys; C!, reduced Cys; M@, oxidized methionine.

The most abundant type of advanced glycation end product (AGE) we identified in the aging heart proteome was carboxymethylation of Lys or Trp, which represents 63% of all peptides with AGEs; this was followed by glyoxal‐derived hydroimidazolone at Arg (23% of peptides with AGEs) (Figure 6a). There were 11 glycated peptides dysregulated in the three conditions, 34 in the two progeric groups, 18 in old WT and *Lmna^G609G^* ^/^ *^G609G^* mice, and 22 in old WT and young *Lmna^G609G^* ^/^ *^wt^* mice (Figure 6b). Enrichment analysis indicated that increased glycation mainly affected proteins related to energy metabolism, cardiac muscle contraction, gene splicing, and vesicle trafficking (Figure 6c,d). The functional categories affected by AGEs thus closely mirrored those affected by reversible oxidation at Cys residues. These results support the occurrence of cardiometabolic disease and oxidative stress in the mouse models of premature and normal aging.

### 2.6 The age‐related protein cardiac alterations in mouse are reproduced in a pig model of progeria

To identify conserved age‐related cardiac changes, we performed quantitative high‐throughput multiplexed proteomic analysis in hearts of WT minipigs and progeroid heterozygous *LMNAc*.*1824C*>*T* minipigs (“HGPS”) (Dorado et al., 2019). Analysis of protein changes in 4 HGPS and 3 WT male pigs identified 3,320 proteins with an FDR<1%, of which 1271 were identified with >1 unique peptide (Table S2). Hierarchical clustering and PCA showed a good separation between progeric and WT animals (Figure S10A,B). Unlike HGPS pigs, WT animals were not bred from the same nuclear donor and therefore show higher dispersion on the PCA plot. Protein abundance changes showed a very good correlation between the different progeric animals (Figure S10D), indicating that HGPS caused reproducible protein alterations in this model.

**FIGURE 7:**
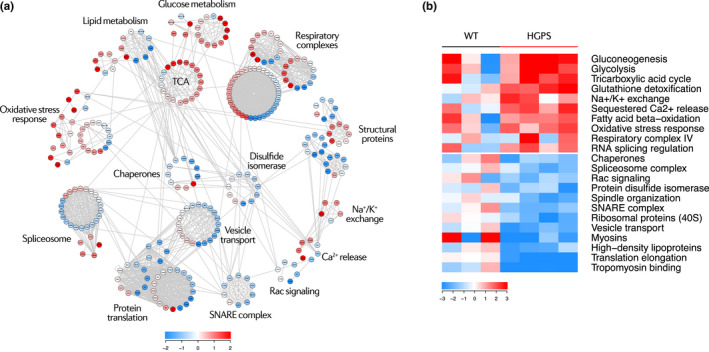
Systems biology overview of the progeric pig heart proteome. (a) Proteomic landscape showing the main categories altered in the progeric pig heart. Each dot represents a protein, color‐coded according to mean relative abundance in the progeric proteome. Color intensity is maximal when |mean Zq| ≥ 2. TCA, tricarboxylic acid. (b) Heatmap of curated categories with altered regulation in the progeric pig model. Selected categories have |Zc| > 1.5 in at least two replicates or |mean Zc|>1.5. Color intensity is maximal when |Zc| ≥ 3.

We found that ~17% of the progeric pig cardiac proteome was altered (Figure S10C). A curated list of differentially expressed proteins is provided as a heat map in Figure S11. SBT analysis revealed coordinated dysregulation of pathways linked to energy metabolism, muscle contraction, gene expression, proteostasis, and response to oxidative stress (Figure 7, Figures S12 and S13). These alterations include upregulation of glucose and fatty acid metabolism (Figure 7, Figure S12A); dysregulation of cytoskeletal proteins, Na^+^/K^+^ channels, and Ca^2+^ signaling (Figure 7, Figure S12B,C); downregulation of protein translation and folding proteins (Figure 7, Figure S13A); dysregulation of RNA splicing mechanisms (Figure 7, Figure S13B); and upregulation of glutathione and other oxidative stress‐response pathways (Figure 7, Figure S13C).

**FIGURE 8:**
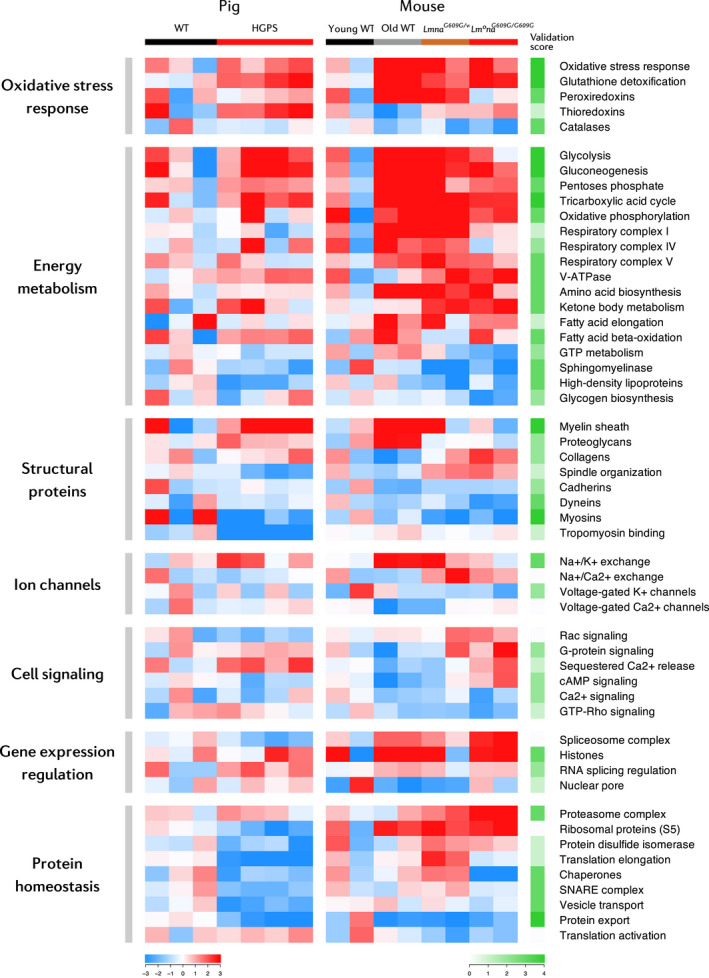
Conserved cardiac changes between mouse and pig aging models. Heatmap of curated categories with altered regulation in premature and/or normal aging mouse and pig models. Selected categories have |Zc| > 1.5 in at least two replicates or |mean Zc| > 1.5. Color intensity is maximal when |Zc| ≥ 3. The validation score measures category change reproducibility between animal models ranging from 0 (non‐reproducible) to 4 (reproducible). This score is the sum in absolute value of the pig and mouse rounded mean Zc (excluding young WT replicates). Mean Zc was rounded to ±2 when |mean Zc| > 1.5 and to ±1 when |mean Zc| < 1.5. Validation score cell intensity is maximal when score = 4.

Following this analysis, we compared the aging mouse and pig heart proteomes, obtaining a set of proteins with conserved altered abundance across species (Figure S14A). These proteins were related to energy metabolism and antioxidant response (Figure S14B). We next analyzed the resemblance between species at the category level, given that the mentioned dysregulated pathways in the progeric pig heart proteome were similar to those found in the progeric or normally aged mouse models. Interestingly, these categories could be classified into the same biological processes and most changed equally in the aging mouse and pig hearts, except for RNA splicing and protein translation pathways (Figure 8). These results suggest that the mechanisms underlying cardiac aging are common to different mammalian species.

## 3 DISCUSSION

Cardiometabolic disease is a hallmark of both normal aging and HGPS. To gain insight into these processes, we examined cardiac and metabolic alterations present in progeric *Lmna^G609G^* mice and young and old WT animals. In agreement with our previous studies (Osorio et al., 2011), we found bradycardia and cardiac electrical abnormalities in *Lmna^G609G^* mice. Compared with young WT animals, old WT and *Lmna^G609G^* ^/^ *^G609G^* mice both present prolonged PQ and QT intervals, as well as T‐wave flattening. In contrast, young *Lmna^G609G^* ^/^ *^wt^* mice present only mild ECG alterations which are aggravated with age, indicating a time‐ and dose‐dependent effect of progerin. These findings are consistent with electrical conduction and repolarization abnormalities that can potentially lead to arrhythmias, which have been previously described for 30‐month‐old WT mice, progeric *Zmpste24*‐deficient mice, HGPS minipigs, and HGPS patients (Dorado et al., 2019; Rivera‐Torres et al., 2016; Signore et al., 2015).

HFpEF is the primary cause of death in HGPS (Gordon, Shappell, et al., 2018). Progression to HFpEF in HGPS patients might result from a combination of diastolic dysfunction, LV hypertrophy, cardiac valve dysfunction, and interstitial myocardial fibrosis (Hanumanthappa, Madhusudan, Mahimarangaiah, & Manjunath, 2011; Hennekam, 2006; Merideth et al., 2008; Nair, Ramachandran, Krishnamoorthy, Dora, & Achuthan, 2004; Prakash et al., 2018), anomalies that are also frequent in the elderly (Biernacka & Frangogiannis, 2011; Horn, 2015; Lakatta, 2003; Wong et al., 2016). Our study reveals that HFpEF could also be fatal to progeric and normally aged mice, since the altered E/A ratio and IVRT in these animals are indicative of diastolic dysfunction. Preserved systolic function and a normal heart mass in all mouse groups suggest an absence of myocardial hypertrophy. Interestingly, compared with young mice, old WT mice have a higher Fulton Index and a higher RV‐to‐LV EDV ratio, which index RV enlargement during normal aging. Collectively, these findings illustrate that the premature and normal aging mouse models accurately recapitulate key aspects of human age‐related heart disease.

Aging is characterized by changes in body composition, with a reduction in bone mineral content (osteopenia) and muscle mass (sarcopenia), as well as a redistribution of fat from subcutaneous to visceral depots, thus eliciting central obesity and peripheral lipodystrophy (Batsis & Villareal, 2018; Tchkonia et al., 2013). These changes are associated with increased risk of other cardiometabolic disorders, such as diabetes, hypertension, atherosclerosis, and dyslipidemia (Shuster, Patlas, Pinthus, & Mourtzakis, 2012). With the exception of obesity, these conditions are present in HGPS patients, although dyslipidemia and (pre)diabetes are not highly prevalent (Gerhard‐Herman et al., 2012; Gordon, Campbell, et al., 2018; Gordon, Harten, Patti, & Lichtenstein, 2005; Merideth et al., 2008). Consistent with these observations, we found that old WT mice become obese, whereas progeric mice develop cachexia and evidence loss of lean mass, together with reduced serum protein and increased serum creatine kinase and transaminases. Reduced HDL levels in blood are a common feature of HGPS patients but are rarely associated with increased plasma LDL or triglyceride (Gerhard‐Herman et al., 2012; Gordon, Campbell, et al., 2018; Gordon et al., 2005; Merideth et al., 2008). Remarkably, serum cholesterol and triglyceride levels are low in *Lmna^G609G^* ^/^ *^G609G^* mice, and *Lmna^G609G^* ^/^ *^wt^* animals also experience a reduction in these markers during disease progression. Similarly, whereas HGPS patients have high insulin levels and some have hyperglycemia and low glucose tolerance (Gerhard‐Herman et al., 2012; Gordon, Campbell, et al., 2018; Gordon et al., 2005; Merideth et al., 2008), progeric mice exhibit hypoglycemia, hypoinsulinemia (López‐Mejía et al., 2014; Osorio et al., 2011), and high glucose tolerance (Figure 2b). We found no alterations in old WT mice except for low glucose levels in non‐fasting conditions. The dissimilarities between HGPS patients and mouse models are unsurprising, as they have been described as interspecific differences between humans and mice (Yin et al., 2012).

Our high‐throughput quantitative proteomic analysis identified more than 7,000 proteins differentially expressed among the normal and premature aging models and young WT mice, outperforming previous proteomic studies on heart tissue during normal aging (Fernandez‐Sanz et al., 2014; Grant et al., 2009; Walther & Mann, 2011). Given the importance of oxidative stress in aging and cardiometabolic disease (Dai et al., 2014; Fernandez‐Sanz et al., 2014; Grant et al., 2009), we analyzed reversible Cys oxidation in the aging mouse heart proteome. We also studied AGEs, posttranslational modifications that affect protein structure and function and accumulate in aging, CVD, and diabetes (Brownlee, 1995; Ruiz‐Meana et al., 2019; Simm, 2013). We provide the first comprehensive list of peptide oxidation and glycation sites that are targeted during aging in the mouse heart, identifying carboxymethylation as the most abundant modification. Most proteins affected by oxidation and glycation were related to metabolic pathways, mitochondria, and cardiac muscle contraction, suggesting that preventing the modification of these residues could potentially ameliorate the cardiometabolic alterations present in HGPS and physiological aging.

To identify protein alterations that are evolutionarily conserved during cardiac aging, we performed a high‐throughput proteomic analysis on hearts from a pig model of progeria and age‐matched WT controls (Dorado et al., 2019), identifying 3,320 proteins. Systems biology analyses of data from our murine and porcine models revealed predominantly HFpEF and aging‐related changes common to all animal models of aging in pathways related to energy metabolism, antioxidant response, cell structure and signaling, gene expression, and proteostasis. Energy metabolism alterations involved increased glucose, amino acid, and fatty acid catabolism, as well as the upregulation of oxidative phosphorylation components. These alterations, as well as the upregulation of the glutathione system and other antioxidant response proteins, are consistent with mitochondrial dysfunction, which has been described in cell and animal models of HGPS (Rivera‐Torres et al., 2013), as well as in the normally aged heart (Dai et al., 2014; Fernandez‐Sanz et al., 2014) and in HFpEF (Kumar, Kelly, & Chirinos, 2019). Ketone body supplementation prolongs health span in old WT mice (Newman et al., 2017), suggesting that the increased ketone body metabolism in prematurely aged mouse and pig hearts reported here may constitute a pro‐survival mechanism. Moreover, ketone bodies might constitute a relevant energy source in HFpEF, as their oxidation might increase cardiac metabolic efficiency (Bedi et al., 2016).

Another hallmark of all our aging heart models is the dysregulation of motor and structural proteins, as well as ion channels and signaling pathways involved in cardiac contraction. Myosins showed decreased relative expression in all mouse and pig aging models, as has been reported in the heart proteome of old monkeys and mice, and in patients with heart failure (Kaushik et al., 2015; Schaper et al., 2002). The downregulation of dyneins, particularly in *Lmna^G609G^* ^/^ *^G609G^* mice, could contribute to mitochondrial dysfunction and loss of proteostasis, as dynein dysfunction disrupts mitochondrial dynamics and autophagosome transport during aging (Bejarano et al., 2018; Eschbach et al., 2013). Cadherins appeared downregulated, more notably in old WT animals, which could compromise intercalated disk integrity and promote arrhythmogenesis in the failing heart (Kostetskii et al., 2005). Conversely, collagens were upregulated in the heart of progeric animals, which is consistent with myocardial fibrosis, present in the progeric pig model (Dorado et al., 2019), in HGPS patients (Hennekam, 2006), in the elderly (Biernacka & Frangogiannis, 2011), and in the failing heart (Piek, de Boer, & Silljé, 2016). Regarding ion channel dysregulation, Na^+^/K^+^ exchangers appeared upregulated in all aging models and more strikingly in old WT mice. This alteration has been reported to promote oxidative stress in the aging heart (Bartlett et al., 2018). Lastly, in relation to signaling pathways, our studies show that calcium, G‐protein, and cAMP signaling proteins are differently dysregulated in the progeric and normal aging models. Through different mechanisms, these alterations may promote calcium mishandling and increase the risk of arrhythmias and failure in progeria and physiological aging (Feridooni, Dibb, & Howlett, 2015; Rivera‐Torres et al., 2016).

Aging is associated with a progressive loss of histones (Sen, Shah, Nativio, & Berger, 2016); however, our proteomic studies reveal increased histone expression in the aged heart. One possible explanation for this apparent discrepancy is that age‐related histone loss is tightly linked to cell division, which is absent in mature cardiomyocytes. Increased histone expression during aging may be an overcompensation mechanism in this tissue. We also detected inverse dysregulation of RNA splicing and protein translation pathways, suggesting disparities in these pathways between progeric pig and mouse hearts. While spliceosome complex components were upregulated in the progeric mouse heart, these proteins were downregulated in the progeric pig model. Nevertheless, RNA splicing regulation is upregulated in both species, suggesting a shared underlying mechanism that may be linked to HFpEF, as spliceosomal components are upregulated in the failing heart (Gao et al., 2011). In relation to protein translation, elongation factors and ribosomal proteins were upregulated in the mouse progeria models and downregulated in the pig model. Translation activation was inversely regulated, suggesting a compensation mechanism. The discrepancy in protein synthesis has been reported in previous studies describing inhibition or promotion of this process (Buchwalter & Hetzer, 2017; Rivera‐Torres et al., 2013). The loss of proteostasis indicated by dysregulation of the proteasome and chaperone systems may play a key role in premature cardiac aging and failure in the mouse and pig progeria models. Chaperone downregulation could lead to the accumulation of unfolded proteins and to an impaired response to stress signals such as oxidative stress; in contrast, proteasome complex upregulation would increase protein clearance in an attempt to compensate the toxic effects elicited by the accumulation of dysfunctional proteins. Overall, the dysregulation of gene expression and protein homeostasis mechanisms and the activation of compensatory systems increase the energy requirements of cardiac cells during aging, which may aggravate the effect of the metabolic alterations detected in this tissue.

The inclusion of the 4‐month‐old *Lmna^G609G^* ^/^ *^wt^* group in our proteomic experiments aimed to assess the impact of progerin dose. At this age, *Lmna^G609G^* ^/^ *^wt^* mice present only mild phenotypical alterations, but they progressively develop conspicuous progeric abnormalities very similar to those found in 4‐month‐old *Lmna^G609G^* ^/^ *^G609G^* mice. Hence, the comparison between 4‐month‐old homozygous and heterozygous animals should help identify early protein changes that might contribute to disease onset and progression. We found that both groups of progeric mice present comparable alterations in categories related to oxidative stress response, energy metabolism, structural proteins, and proteostasis, thus identifying changes in cardiac proteins that might initiate and sustain cardiac disease in progeria. Changes in cell signaling and gene expression regulation were less obvious in young heterozygous animals, indicating that these processes are affected only at advanced stages of the disease. Interestingly, some protein groups, as mitochondrial complexes, peroxiredoxins, ion exchangers, or chaperones, appeared more clearly upregulated in young *Lmna^G609G^* ^/^ *^wt^* mice than in age‐matched *Lmna^G609G^* ^/^ *^G609G^* animals, which could indicate a switch in regulation during disease progression.

Our comparative analysis of the proteomes of mouse and pig aging models identified a set of proteins and pathways that are dysregulated in an evolutionarily conserved manner. Although future research is needed to confirm causal relationships between these alterations and age‐related cardiac dysfunction, these candidates could be targeted to develop rejuvenation or preventive strategies in the setting of normal aging and HGPS. Our results identify cardiac and metabolic alterations that are common in physiological and premature aging models and mammalian species. This highlights the relevance of using animal models of HGPS to study age‐related human diseases and ultimately to promote healthier aging in the general population.

## 4 EXPERIMENTAL PROCEDURES

Detailed material and methods are provided in the Supplementary data online.

### 4.1 Animal care

Mouse studies were carried out in *Lmna^G609G^* animals (Osorio et al., 2011) and in WT mice on the C57BL/6 genetic background. Pig proteomics was performed with WT and *LMNAc*.*1824C*>*T* Yucatan minipigs (Dorado et al., 2019). All experimental procedures were performed in accordance with EU Directive 2010/63EU and Recommendation 2007/526/EC, enforced in Spanish law under Real Decreto 53/2013.

### 4.2 Heart rate

HR was measured with a non‐invasive automated tail‐cuff device (BP2000, Visitech Systems) in conscious mice to avoid anesthetic interference. Measurements were taken on three consecutive days without applying tail‐cuff pressure.

### 4.3 Electrocardiography

ECG recordings were acquired at 2‐KHz sweep speed using a MP36R data acquisition workstation (BIOPAC Systems). ECG data were exported with AcqKnowledge software (BIOPAC Systems) and automatically analyzed using custom R scripts.

### 4.4 Echocardiography

Transthoracic echocardiography was performed using a high‐frequency ultrasound system (Vevo 2100, VisualSonics) with a 40‐MHz linear probe. Two‐dimensional (2D) echography and M‐mode echography were performed at a frame rate >230 frames/s, and pulsed wave (PW) Doppler was acquired with a pulse repetition frequency of 40 kHz.

### 4.5 Magnetic resonance imaging

MRI studies were performed with a 7‐T Agilent/Varian scanner (Agilent Technologies) equipped with a DD2 console. Cardiac MRI was performed using an actively shielded 115/60 gradient. Body fat images were acquired using a spin‐echo multi‐slice (SEMS) sequence with and without fat. Cine MRI was analyzed using the freely available Segment software (Heiberg et al., 2010). Whole‐body images were segmented using Advances Normalization Tools (Avants et al., 2011).

### 4.6 Tolerance to challenge tests and blood analysis

For GTT, overnight fasted mice received an intraperitoneal injection of glucose (1 g/kg body weight, Sigma‐Aldrich). In ITT studies, 1‐hr fasted mice received an intraperitoneal injection of insulin (0.75 IU/kg, Lilly). Blood glucose levels were determined in tail‐vein blood at 0, 15, 30, 60, 90, and 120 min post‐injection with a Contour Next One Smart Meter (Contour Next). For biochemical analysis, animals were fasted overnight before blood was extracted. Biochemical variables were analyzed using a Dimension RxL Max Integrated Chemistry System (Siemens Healthineers).

### 4.7 Sample preparation for proteomics

Mouse and pig heart samples were collected at necropsy and snap‐frozen in liquid nitrogen. Protein extracts were obtained by homogenizing tissue in lysis buffer and samples were subjected to tryptic digestion using filter‐aided sample preparation (FASP) technology (Expedeon), which for mouse samples was adapted for oxidized Cys labeling (Bonzon‐Kulichenko et al., 2020). Eluted and cleaned‐up peptides were subjected to 8plex isobaric labeling (iTRAQ, AB Sciex).

### 4.8 Liquid chromatography coupled to tandem mass spectrometry

The tryptic peptide mixtures were subjected to nano‐LC‐MS/MS on an EASY‐nLC 1000 liquid chromatograph (Thermo Fisher Scientific) coupled to either an Orbitrap Fusion mass spectrometer or a Q Exactive HF mass spectrometer (Thermo Fisher Scientific).

### 4.9 Quantitative analysis of proteomic data

Proteins were identified using the SEQUEST HT algorithm integrated into Proteome Discoverer 2.1 (Thermo Fisher Scientific). SEQUEST results were analyzed by the probability ratio and refined methods (Bonzon‐Kulichenko, García‐Marqués, Trevisan‐Herraz, & Vázquez, 2015; Martínez‐Bartolomé et al., 2008). Comparative analysis of protein abundance changes was conducted with the SanXoT software package (Trevisan‐Herraz et al., 2019) based on the Weighted Scan‐Peptide‐Protein (WSPP) statistical model (Navarro et al., 2014). For the study of coordinated protein alterations, we used the Systems Biology Triangle (SBT) model (García‐Marqués et al., 2016).

### 4.10 Statistical analysis and data visualization

Experimental conditions were randomized, and comparisons made between age‐matched groups including male and female mice. Statistical analysis of the results was performed with custom R scripts that apply multifactorial logistic or linear models to compare groups. Significant differences were considered when *p* < 0.05. Results were represented in R as beeswarm boxplots or scatter plots with linear or LOESS curve fitting. Proteomic networks were interpreted with Cytoscape (The Cytoscape Consortium).

## CONFLICT OF INTEREST

The authors have no conflict of interest to declare.

## AUTHOR CONTRIBUTIONS

V.F. carried out experimental work, data analysis and interpretation, and manuscript preparation. I.J. and E.C. performed data analysis and participated in manuscript preparation. A.M., C.G.‐G., A.B., and M.J.A.M. performed experimental work. B.D. and J.R.‐T. provided critical materials and insights. J.V., C.L.‐O., and V.A. supervised project planning and development, and commented on the manuscript at all stages.

## Supporting information

Figures S1‐S14

Table S1

Table S2

## Data Availability

Data that support the findings of this study are available from the corresponding authors upon request. The mass spectrometry proteomic data have been deposited to the ProteomeXchange Consortium via the PRIDE partner repository with the dataset identifiers PXD019532 and PXD019533.
