# Supplementary material for: Identification of common cardiometabolic alterations and deregulated pathways in mouse and pig models of aging
Source: Aging Cell. 2020 Jul 30;19(9):e13203. doi: 10.1111/acel.13203 (PMC7511870; doi:10.1111/acel.13203)
Supplement: Supplementary file 1 — Figures S1‐S14 [file ACEL-19-e13203-s001.pdf]

Supplementary Material

Identification of common cardiometabolic alterations and deregulated pathways in mouse and pig models of aging

Víctor Fanjul [1,2,3], Inmaculada Jorge [1,3], Emilio Camafeita [1,3], Álvaro Macías[1,3], Cristina González-Gómez [1,3], Ana Barettino [1,3], Beatriz Dorado [1,3], María Jesús Andrés-Manzano [1,3], José Rivera-Torres [1,3,+], Jesús Vázquez [1,3], Carlos López-Otín [2,4,*], Vicente Andrés [1,3,*]

1. Centro Nacional de Investigaciones Cardiovasculares Carlos III (CNIC), Madrid 28029, Spain. 2. Departamento de Bioquímica y Biología Molecular, Facultad de Medicina, Instituto Universitario de Oncología, Universidad de Oviedo, 33006 Oviedo, Spain. 3. Centro de Investigación Biomédica en Red Enfermedades Cardiovasculares (CIBERCV), Spain. 4. Centro de Investigación Biomédica en Red Enfermedades Cáncer (CIBERONC), Spain.

*Co-corresponding authors: Vicente Andrés (vandres@cnic.es); Carlos López-Otín (clo@uniovi.es)

+ Current address: Facultad de Ciencias Biomédicas y de la Salud, Universidad Europea de Madrid UEM), Spain.

Content

Supplementary Experimental Procedures

Supplementary References

Supplementary Tables (1-2)

Supplementary Figures (1-14)

Supplementary Experimental Procedures

Animal Care. Mouse studies were carried out in 4-month-old progeric *Lmna*$^{G609G/G609G}$ and *Lmna*$^{G609G/wt}$ animals (Osorio et al., 2011) and in 4-month-old and 20-month-old wild-type (WT) mice on the C57BL/6 genetic background. When indicated, we used 10-month-old *Lmna*$^{G609G/wt}$ and WT mice. Unless otherwise specified, experiments were performed with a balanced proportion of male and female mice to avoid gender-biased results. Pig proteomics was performed with castrated ~5-month-old male heterozygous *LMNAc.1824C>T* Yucatan minipigs (Dorado et al., 2019) or WT Yucatan minipigs. All experimental procedures with mice and pigs were reviewed and authorized by Ethics Committees (CAM-PROEX 313/16 and PROEX 216/19) in accordance with EU Directive 2010/63EU and Recommendation 2007/526/EC, enforced in Spanish law under Real Decreto 53/2013. Every effort was made to minimize animal suffering.

Heart Rate. Heart rate (HR) was measured with a non-invasive automated tail-cuff device (BP2000, Visitech Systems) in conscious mice to avoid anesthetic interference. Measurements were taken on three consecutive days without applying tail-cuff pressure. To improve accuracy, the first 10 of 20 measurements were discarded. The interquartile range (IQR) was calculated (difference between quartiles 3 and 1), and data points 1.5xIQR below the first quartile (Q1) or 1.5xIQR above the third quartile (Q3) were discarded as outliers.

Electrocardiography. Mice were anesthetized with 1-2% isoflurane, and 4 electrocardiography (ECG) electrodes were inserted subcutaneously into the limbs. ECG recordings were acquired for 2 min at 2 KHz sweep-speed using a MP36R data acquisition workstation (Biopac Systems). ECG data were exported with AcqKnowledge software (Biopac Systems) and automatically analyzed using custom R scripts, which remove artifacts, noise and baseline fluctuations; detect heart beats, peaks and waves; and calculate ECG intervals. The second lead was selected for the study, since the signal was more stable in most experiments, and therefore the identification of waves was more robust using this channel.

PQ interval was measured from the P-wave peak to the beginning of the Q-wave (Q_s). The end of the S-wave (J) is not evident in mice because the ST segment is absent in this species; therefore, the QRS complex was calculated from the Q_s to the S-wave minimum, and the QT interval was calculated from the Q_s to the T-wave peak. The QT_{90} was defined as the interval between the Q_s and the point where the T-wave declines by 90% (T_{90}) from the peak. QT_e was also measured, from Q_s to the point where the negative component of the T-wave returned to the isoelectric line (T_e). T-wave morphological alterations were quantified by defining the JT gap and the T-wave steepness. The JT gap measures the distance between the J point and the T-wave peak, typically coincidental in mice, thus allowing detection of delays in T-wave appearance. T-wave steepness serves as a measure of T-wave flattening because it represents the absolute value of the slope (difference in voltage divided by the difference in time) between the peak of the T-wave and T_{90}.

Echocardiography. Transthoracic echocardiography was performed by an expert operator blinded to genotype using a high- frequency ultrasound system (Vevo 2100, VisualSonics) with a 40 MHz linear probe. Before echocardiography, animal fur was removed with a topical depilatory agent. Two-dimensional (2D) and M-mode echography were performed at a frame rate >230 frames/s and pulsed wave (PW) Doppler was acquired with a pulse repetition frequency of 40 kHz. Mice were lightly anesthetized with 1-2% isoflurane in oxygen through a facial mask, adjusting the isoflurane delivery to maintain the HR at 450 $\pm$ 50 bpm. Mice were placed in a supine position on a heating platform, and warmed ultrasound gel was used to maintain normothermia. A base apex ECG was continuously monitored. Images were analyzed off-line using the Vevo 2100 Workstation software (VisualSonics).

For left ventricle (LV) systolic function assessment, parasternal standard 2D and M-mode, long axis (LAX) and short axis (SAX) views were acquired (Moran et al., 2013). LV chamber dimensions were measured from these views, which served to calculate LV ejection fraction (EF) and LV chamber mass (Foppa et al., 2005). Cardiac Output (CO) was calculated by measuring HR, the area of the LV outflow tract diameter (LVOTd) and the velocity time integral (VTI) (Zoghbi & Quinones, 1986). LVOTd was assessed from a 2D LAX view, at the level of the aortic valve annulus, after maximal systolic leaflet separation. VTI was obtained by tracing the LVOT flow signal envelope, which was acquired by aligning the PW Doppler beam with the LV outflow. Mitral Valve (MV) inflow pattern was acquired using PW Doppler echography in the 4-chamber apical view to assess diastolic function. The sample volume was positioned parallel to the blood flow, across the mitral orifice. Measured variables included early and late diastolic velocity peak wave (E and A, respectively), the E/A ratio and isovolumetric relaxation time (IVRT) (Schnelle et al., 2018).

Magnetic Resonance Imaging. Magnetic resonance imaging (MRI) studies were performed at the CNIC Advanced Imaging Unit with a 7-T Agilent/Varian scanner (Agilent Technologies) equipped with a DD2 console. For image acquisition, mice were sedated with 2% isoflurane in oxygen. Ophthalmic gel was applied to prevent retinal drying. Cardiac MRI was performed using an actively shielded 115/60 gradient. A surface coil was used for MRI signal transmission and reception. MRI experiments were conducted by applying an ECG-triggered fast gradient echo cine sequence with the following imaging parameters: repetition time, 125 ms (minimum values depending on HR and number of frames per heart cycle); echo time, 1.25 ms; field of view, 30 x 30 mm; acquisition matrix, 128 x 128; flip angle, 15º; 4 averages; 20 cardiac phases; and 12 0.8 mm slices (depending on heart size) with a 0.2 mm gap.

The examination began with the acquisition of survey chest images in the three orthogonal planes (transverse, coronal and sagittal) to localize the heart and obtain images in two-chamber SAX view, 4-chamber view and two-chamber LAX view. By using the 4-chamber and two-chamber LAX views, a series of parallel SAX images were defined (encompassing the entire LV from the base to the apex). The apical-most slice of the multislice set was positioned inferior to the heart, and the basal-most slice was positioned above the LV to ensure total coverage of the ventricles through the cardiac cycle. Body fat images were acquired using a spin echo multi slice (SEMS) sequence with and without fat saturation and with the following parameters: repetition time, 533-884 ms (varies between with and without fat saturation); echo time, 10.44 ms; acquisition matrix, 128 x 128; flip angle, 90º; 1 average; and 35 1 mm slices with a 1 mm gap.

Cine MRI was analyzed using the freely available Segment software (Heiberg et al., 2010). On both ventricles, epicardial and endocardial borders were delineated manually on the SAX of each slide to measure total ventricular volumes and cardiac mass (Hernández-Porras et al.,

2014). Cardiac function was assessed by calculating LV and right ventricle (RV) EF, stroke volume (SV) and CO from these measurements. Additionally, the RV to LV end-diastolic volume (EDV) ratio and the Fulton index (RV/LV mass) were determined to analyze ventricle relative size. Whole body images were first bias-field-corrected and segmented using Advances Normalization Tools (Avants et al., 2011). The partial volume estimate corresponding to fat was then masked in order to remove brain tissue from whole body fat composition, and the volume of non-zero voxels was calculated in FSL (Jenkinson et al., 2012). Brain masks were manually drawn in ITK snap (Yushkevich et al., 2006). In cases requiring manual correction of the fat segmentation images, partial volumes were intensity-rescaled and re-segmented. Fat mass was estimated as the product of fat volume and fat density (0.923 g/cm^3) (Hill et al., 2007).

Tolerance to Challenge Tests and Blood Analysis. For glucose tolerance tests (GTT), overnight fasted mice received an intraperitoneal injection of glucose (1 g/kg body weight, Sigma-Aldrich). In insulin tolerance tests (ITT) studies, 1 h fasted mice received an intraperitoneal injection of insulin (0.75 IU/kg, Lilly). Blood glucose levels were determined in tail-vein blood at 0, 15, 30, 60, 90 and 120 min post injection with a Contour Next One Smart Meter (Contour Next).

For biochemical analysis, animals were fasted overnight before blood was extracted directly from the mandibular sinus (live mice) or from the heart and renal artery (euthanized mice). Blood samples were collected in plastic tubes and incubated at room temperature for 1 to 2 h to allow clotting. Serum samples were later centrifuged at 1200 g for 15 min and the supernatant was stored at -80 ºC. Biochemical variables were analyzed using a Dimension RxL Max Integrated Chemistry System (Siemens Healthineers).

Sample Preparation for Proteomics. Mice were euthanized in a CO_2 chamber, and 0.9% saline-perfused hearts were collected, snap-frozen in liquid nitrogen, pulverized with a pestle and mortar and preserved at -80 ºC until use. Protein extracts were obtained by homogenizing tissue with ceramic beads (MagNa Lyser Green Beads apparatus, Roche) in lysis buffer (1.5% SDS, 1 mM EDTA, 50 mM Tris-HCl at pH 8.5) supplemented with 50 mM iodoacetamide (Sigma-Aldrich) to block reduced Cys residues (Martínez-Acedo et al., 2012). Protein concentrations were determined by RC/DC Protein Assay (Bio-Rad Laboratories). We analyzed two samples for each experimental condition, each sample consisting of a pool of 2 hearts, ensuring equal contributions from each biological replicate.

Pig samples were small portions of ventricular tissue obtained at necropsy and snap-frozen in liquid nitrogen. Samples were suspended in lysis buffer (50 mM Tris HCl pH 7.5, 150 mM NaCl, 0.1% SDS, 0.5% sodium deoxycholate, 1% NP-40, 1 mM DTT) containing protease and phosphatase inhibitors (Roche), and protein extracts were obtained by mechanical disruption with POLYTRON® PT 6100 homogenizers (Kinematica) and sonication with a Bioruptor (Diagenode) at 4 ºC.

Samples were subjected to tryptic digestion using filter-aided sample preparation (FASP) technology (Expedeon) according to the previously published method (Wiśniewski et al., 2009), which for mouse samples was adapted for oxidized Cys labeling based on the FASILOX method (Bonzon-Kulichenko et al., 2020). Briefly, protein extracts (100 µg) were diluted in urea sample solution (USS, 8 M urea in 100 mM Tris-HCl at pH 8.5) and loaded onto the filters. After centrifugation and a washing step with USS, Cys residues were reduced with 50 mM DTT (GE Healthcare) in preparation buffer (50 mM HEPES, 1 mM EDTA at pH 7.5) for 1 h at room temperature. Next, the samples were centrifuged, washed with preparation buffer and subsequently alkylated with 50 mM S-methyl methanethiosulfonate (MMTS) (Thermo Fisher

Scientific) in preparation buffer for 1 h at room temperature. Filters were then washed three times with USS and three times with ABC Buffer (50 mM NH_4HCO_3 at pH 8.8). Proteins were digested using sequencing grade trypsin (Promega) at a 1:40 trypsin: sample-mass ratio.

Eluted and cleaned-up peptides were subjected to isobaric tag for relative and absolute quantitation (iTRAQ) 8-plex labeling (AB Sciex), combined and desalted on Waters Oasis HLB C18 cartridges (Waters Corp). Labeled mouse peptides were separated into eight fractions using Waters Oasis MCX cartridges (Waters Corp) and graded concentrations of ammonium formate (pH 3.0) (AF3) in acetonitrile (ACN). Briefly, desalted and dried peptides were taken up in 1 ml of 5 mM AF3 containing 25% (v/v) ACN. The MCX cartridge was equilibrated with 1 ml of 1:1 methanol:water, followed by 3 ml of 5 mM AF3 containing 25% (v/v) ACN. After loading the samples, the cartridge was washed with AF3, 25% (v/v) ACN. Bound peptides were eluted with the following solvents: 1) 500 mM AF3, 25% (v/v) ACN; 2) 1 M AF3, 25% (v/v) ACN; 3) 1.5 M AF3, 25% (v/v) ACN; 4) 500 mM AF3, 25% (v/v) ACN, 1 M KCl; 5) 1.25 M AF3, 37,5% (v/v) ACN; 6) 1.25 M AF3, 37,5% (v/v) ACN; 7) 1 M AF3, 50% (v/v) ACN; and 8) 1 M AF3, 50% (v/v) ACN. The fractions obtained were purified and desalted with the MiniSpin Column Kit (The Nest Group), dried and stored at -20 ºC until use. Labeled pig peptides were separated into five fractions using the high pH reversed-phase peptide fractionation kit (Thermo Fisher Scientific). Bound peptides were eluted with the following solvents: 1) 12.5% (v/v) ACN; 2) 15% (v/v) ACN; 3) 17.5% (v/v) ACN; 4) 20% (v/v) ACN; and 5) 50% ACN. The fractions obtained were vacuum-dried and stored at -20 ºC until use.

Liquid Chromatography Coupled to Tandem Mass Spectrometry. The tryptic peptide mixtures were subjected to nanoscale liquid chromatography (LC) coupled to tandem mass spectrometry (MS/MS). High-resolution analysis of samples was performed on an EASY-nLC 1000 liquid chromatograph (Thermo Fisher Scientific) coupled to either an Orbitrap Fusion mass spectrometer (Thermo Fisher Scientific) or a Q Exactive HF mass spectrometer (Thermo Fisher Scientific). Peptides were suspended in 0.1% formic acid and loaded onto a C18 RP nano-precolumn (Acclaim PepMap100: internal diameter, 75 µm; particle size, 3 µm; and length, 2 cm; Thermo Fisher Scientific). Peptides were then separated on an analytical C18 nano-column (EASY-Spray column PepMap RSLC C18: internal diameter 75 µm; particle size 3 µm; and length, 50 cm; Thermo Fisher Scientific) in a continuous gradient: 8-27% B for 240 min, 31-100% B for 2 min, 100% B for 7 min, 100-2% B for 2 min and 2% B for 30 min (where A is 0.1% formic acid in HPLC-grade water and B is 90% ACN, 0.1% formic acid in HPLC-grade water).

Spectra were acquired using full ion-scan mode over the 400-1,500 mass-to-charge (m/z) range and 120,000 resolution. The automatic gain control target was set at 2 x 10^5 with a maximum injection time of 50 ms. Data-dependent speed mode acquisition was performed at 5 x 10^4 automatic gain control and 120 ms injection time, with a 1 Da isolation window and 45 s dynamic exclusion. High collision energy dissociation fragmentation was set to 33% normalized collision energy. MS/MS scan resolution was set to 15,000, and the first mass in the fragmentation spectrum range was fixed at 100 m/z.

Quantitative Analysis of Proteomic Data. Proteins were identified in the raw files using the SEQUEST HT algorithm integrated in Proteome Discoverer 2.1 (Thermo Fisher Scientific). MS/MS scans were matched against protein databases for the mouse (UniProtKB 2016-07 release) or for the pig (UniProtKB 2018-09 release) supplemented with pig trypsin and human keratin sequences. Database search parameters were as follows: trypsin digestion with maximum 2 missed cleavage allowed, precursor mass tolerance of 800 ppm and a fragment mass tolerance of 0.02 Da. Fixed modifications were the N-terminal and lysine iTRAQ 8-plex modifications, whereas variable modifications were methionine oxidation, Cys carbamidomethylation and Cys methylthiolation. To study AGE posttranslational modifications, several AGEs described in the Unimod database (www.unimod.org) were selected as additional variable modifications: glyoxal-derived hydroimidazolone, methylglyoxal-derived hydroimidazolone, malondialdehyde adduct, dihydroxyimidazolidine, hydroxyphenylglyoxal, phenylglyoxal adduct and bis(hydroxyphenylglyoxal) in arginine; malondialdehyde adduct, 2-ammonio-6-[4-(hydroxymethyl)-3-oxidopyridinium-1-yl]-hexanoate and carboxymethylation in lysine; and tryptophan carboxymethylation. SEQUEST results were analyzed by the probability ratio (pRatio) method (Martínez-Bartolomé et al., 2008). Calculation of the false discovery rate (FDR) was based on the results obtained by database searching against the corresponded inverted database using the refined method (Bonzon-Kulichenko et al., 2015; Navarro & Vázquez, 2009). Quantitative information was extracted from the intensity of iTRAQ reporter ions in the low-mass region of the 3MS/MS spectra (García-Marqués et al., 2016).

Comparative analysis of protein abundance changes was conducted with the SanXoT software package (Trevisan-Herraz et al., 2019), which was designed for the statistical analysis of high-throughput, quantitative proteomics experiments and is based on the Weighted Scan-Peptide-Protein (WSPP) statistical model (Navarro et al., 2014). As input, WSPP uses a list of quantifications in the form of $\log_2$-ratios (each condition versus the mean for control 4-month-old WT mice or WT pigs) with their statistical weights. From these, WSPP generates the standardized forms of the original variables by computing the quantitative values expressed in units of standard deviation around the means (Zq). Proteins quantified with only 1 unique peptide were excluded from further analysis. Within-group and between-group variability was assessed in the samples by fitting unsupervised Machine Learning models (hierarchical clustering and principal component analysis: PCA) and by pairwise correlations between replicates and between conditions. Unless otherwise indicated, correlation tests and subsequent analyses were performed to proteins with a between-replicate absolute mean Z score higher than 1.5 ($|\text{mean Zq}|>1.5$) in at least one condition. Known artifact proteins such as keratin contaminants, trypsin, and several major serum proteins (albumin, globins, serpins and complement factors) were excluded from the datasets after the analysis.

For the study of coordinated protein alterations, we used the Systems Biology Triangle (SBT) model, which estimates functional category averages (Zc) from protein values by performing the protein-to-category integration (García-Marqués et al., 2016). The protein category database was consolidated by including resources from Ingenuity Pathway Analysis (IPA) (Qiagen), CORUM (Helmholtz Zentrum München) and DAVID (Leidos Biomedical Research) (Gene Ontology - GO, KEGG Pathway, BioCarta, InterPro, PIR SuperFamily, SMART). Protein interaction networks were analyzed with STRING (ELIXIR Core Data Resources).

Statistical Analysis and Data Visualization. Experimental conditions were randomized and comparisons in animal studies were made between age-matched groups including male and female mice. No statistical method was used to predetermine sample size. Statistical analysis of the results was performed with custom R scripts that apply multifactorial logistic, linear or generalized linear models to compare groups. Natural-logarithm transformations were considered when the normality condition was not met. Significant differences were considered when $p<0.05$. The following factors were considered as fixed effects: condition (genotype, age group), sex, weight (MRI) and time (tolerance tests). Sample replicates (HR) and date of measurement were included in the models as random effects. Results were represented in R as beeswarm-boxplots or scatter plots with linear or LOESS curve fitting. Boxes in boxplots indicate quartiles and median; whiskers represent the 1.5xIQR; and non-shaded dots are candidate outliers. The shaded area in scatter plots corresponds to the 95% confidence interval of the LOESS curve. Proteomic networks were interpreted with Cytoscape (The Cytoscape Consortium).

Supplementary References

Avants, B. B., Tustison, N. J., Song, G., Cook, P. A., Klein, A., & Gee, J. C. (2011). A reproducible evaluation of ANTs similarity metric performance in brain image registration. *NeuroImage*, *54*(3), 2033–2044. https://doi.org/10.1016/j.neuroimage.2010.09.025

Bonzon-Kulichenko, E., Camafeita, E., López, J. A., Gómez-Serrano, M., Jorge, I., Calvo, E., Núñez, E., Trevisan-Herraz, M., Bagwan, N., Bárcena, J. A., Peral, B., & Vázquez, J. (2020). Improved integrative analysis of the thiol redox proteome using filter-aided sample preparation. *Journal of Proteomics*, *214*, 103624. https://doi.org/10.1016/j.jprot.2019.103624

Bonzon-Kulichenko, E., García-Marqués, F., Trevisan-Herraz, M., & Vázquez, J. (2015). Revisiting Peptide Identification by High-Accuracy Mass Spectrometry: Problems Associated with the Use of Narrow Mass Precursor Windows. *Journal of Proteome Research*, *14*(2), 700–710. https://doi.org/10.1021/pr5007284

Dorado, B., Pløen, G. G., Barettino, A., Macías, A., Gonzalo, P., Andrés-Manzano, M. J., González-Gómez, C., Galán-Arriola, C., Alfonso, J. M., Lobo, M., López-Martín, G. J., Molina, A., Sánchez-Sánchez, R., Gadea, J., Sánchez-González, J., Liu, Y., Callesen, H., Filgueiras-Rama, D., Ibáñez, B., … Andrés, V. (2019). Generation and characterization of a novel knockin minipig model of Hutchinson-Gilford progeria syndrome. *Cell Discovery*, *5*(1), 16. https://doi.org/10.1038/s41421-019-0084-z

Foppa, M., Duncan, B. B., & Rohde, L. E. P. (2005). Echocardiography-based left ventricular mass estimation. How should we define hypertrophy? *Cardiovascular Ultrasound*, *3*, 17. https://doi.org/10.1186/1476-7120-3-17

García-Marqués, F., Trevisan-Herraz, M., Martínez-Martínez, S., Camafeita, E., Jorge, I., Lopez, J. A., Méndez-Barbero, N., Méndez-Ferrer, S., Del Pozo, M. A., Ibáñez, B., Andrés, V., Sánchez-Madrid, F., Redondo, J. M., Bonzon-Kulichenko, E., & Vázquez, J. (2016). A novel systems-biology algorithm for the analysis of coordinated protein responses using quantitative proteomics. *Molecular & Cellular Proteomics*, *15*(5), 1740–1760. https://doi.org/10.1074/mcp.M115.055905

Heiberg, E., Sjögren, J., Ugander, M., Carlsson, M., Engblom, H., & Arheden, H. (2010). Design and validation of Segment - freely available software for cardiovascular image analysis. *BMC Medical Imaging*, *10*(1), 1. https://doi.org/10.1186/1471-2342-10-1

Hernández-Porras, I., Fabbiano, S., Schuhmacher, A. J., Aicher, A., Cañamero, M., Cámara, J. A., Cussó, L., Desco, M., Heeschen, C., Mulero, F., Bustelo, X. R., Guerra, C., & Barbacid, M. (2014). K-Ras V14I recapitulates Noonan syndrome in mice. *Proceedings of the National Academy of Sciences*, *111*(46), 16395–16400. https://doi.org/10.1073/pnas.1418126111

Hill, A. M., LaForgia, J., Coates, A. M., Buckley, J. D., & Howe, P. R. C. (2007). Estimating abdominal adipose tissue with DXA and anthropometry. *Obesity*, *15*(2), 504–510. https://doi.org/10.1038/oby.2007.629

Jenkinson, M., Beckmann, C. F., Behrens, T. E. J., Woolrich, M. W., & Smith, S. M. (2012). FSL. *NeuroImage*, *62*(2), 782–790. https://doi.org/10.1016/j.neuroimage.2011.09.015

Martínez-Acedo, P., Núñez, E., Gómez, F. J. S., Moreno, M., Ramos, E., Izquierdo-Álvarez, A., Miró-Casas, E., Mesa, R., Rodriguez, P., Martínez-Ruiz, A., Dorado, D. G., Lamas, S., & Vázquez, J. (2012). A novel strategy for global analysis of the dynamic thiol redox proteome. *Molecular & Cellular Proteomics*, *11*(9), 800–813. https://doi.org/10.1074/mcp.M111.016469

Martínez-Bartolomé, S., Navarro, P., Martín-Maroto, F., López-Ferrer, D., Ramos-Fernández, A., Villar, M., García-Ruiz, J. P., & Vázquez, J. (2008). Properties of average score distributions of SEQUEST: the probability ratio method. *Molecular & Cellular Proteomics*, *7*(6), 1135–1145.

https://doi.org/10.1074/mcp.M700239-MCP200

Moran, C. M., Thomson, A. J. W., Rog-Zielinska, E., & Gray, G. A. (2013). High-resolution echocardiography in the assessment of cardiac physiology and disease in preclinical models. *Experimental Physiology*, *98*(3), 629–644. https://doi.org/10.1113/expphysiol.2012.068577

Navarro, P., Trevisan-Herraz, M., Bonzon-Kulichenko, E., Núñez, E., Martínez-Acedo, P., Pérez-Hernández, D., Jorge, I., Mesa, R., Calvo, E., Carrascal, M., Hernáez, M. L., García, F., Bárcena, J. A., Ashman, K., Abian, J., Gil, C., Redondo, J. M., & Vázquez, J. (2014). General statistical framework for quantitative proteomics by stable isotope labeling. *Journal of Proteome Research*, *13*(3), 1234–1247. https://doi.org/10.1021/pr4006958

Navarro, P., & Vázquez, J. (2009). A refined method to calculate false discovery rates for peptide identification using decoy databases. *Journal of Proteome Research*, *8*(4), 1792–1796. https://doi.org/10.1021/pr800362h

Osorio, F. G., Navarro, C. L., Cadiñanos, J., López-Mejía, I. C., Quirós, P. M., Bartoli, C., Rivera-Torres, J., Tazi, J., Guzmán-Martínez, G., Varela, I., Depetris, D., de Carlos, F., Cobo, J., Andrés, V., De Sandre-Giovannoli, A., Freije, J. M. P., Lévy, N., & López-Otín, C. (2011). Splicing-directed therapy in a new mouse model of human accelerated aging. *Science Translational Medicine*, *3*(106), 106ra107. https://doi.org/10.1126/scitranslmed.3002847

Schnelle, M., Catibog, N., Zhang, M., Nabeebaccus, A. A., Anderson, G., Richards, D. A., Sawyer, G., Zhang, X., Toischer, K., Hasenfuss, G., Monaghan, M. J., & Shah, A. M. (2018). Echocardiographic evaluation of diastolic function in mouse models of heart disease. *Journal of Molecular and Cellular Cardiology*, *114*, 20–28. https://doi.org/10.1016/J.YJMCC.2017.10.006

Trevisan-Herraz, M., Bagwan, N., García-Marqués, F., Rodriguez, J. M., Jorge, I., Ezkurdia, I., Bonzon-Kulichenko, E., & Vázquez, J. (2019). SanXoT: a modular and versatile package for the quantitative analysis of high-throughput proteomics experiments. *Bioinformatics*, *35*(9), 1594–1596. https://doi.org/10.1093/bioinformatics/bty815

Wiśniewski, J. R., Zougman, A., Nagaraj, N., & Mann, M. (2009). Universal sample preparation method for proteome analysis. *Nature Methods*, *6*(5), 359–362. https://doi.org/10.1038/nmeth.1322

Yushkevich, P. A., Piven, J., Hazlett, H. C., Smith, R. G., Ho, S., Gee, J. C., & Gerig, G. (2006). User-guided 3D active contour segmentation of anatomical structures: Significantly improved efficiency and reliability. *NeuroImage*, *31*(3), 1116–1128. https://doi.org/10.1016/j.neuroimage.2006.01.015

Zoghbi, W. A., & Quinones, M. A. (1986). Determination of cardiac output by Doppler echocardiography: a critical appraisal. *Herz*, *11*(5), 258–268. http://www.ncbi.nlm.nih.gov/pubmed/3781460

Supplementary Tables

Supplementary tables are provided as Excel files. In this document we include the table legends:

Supplementary Table 1. Relative protein abundance in the aging mouse heart proteome.

Supplementary Table 2. Relative protein abundance in the progeric pig heart proteome.

Supplementary Figures

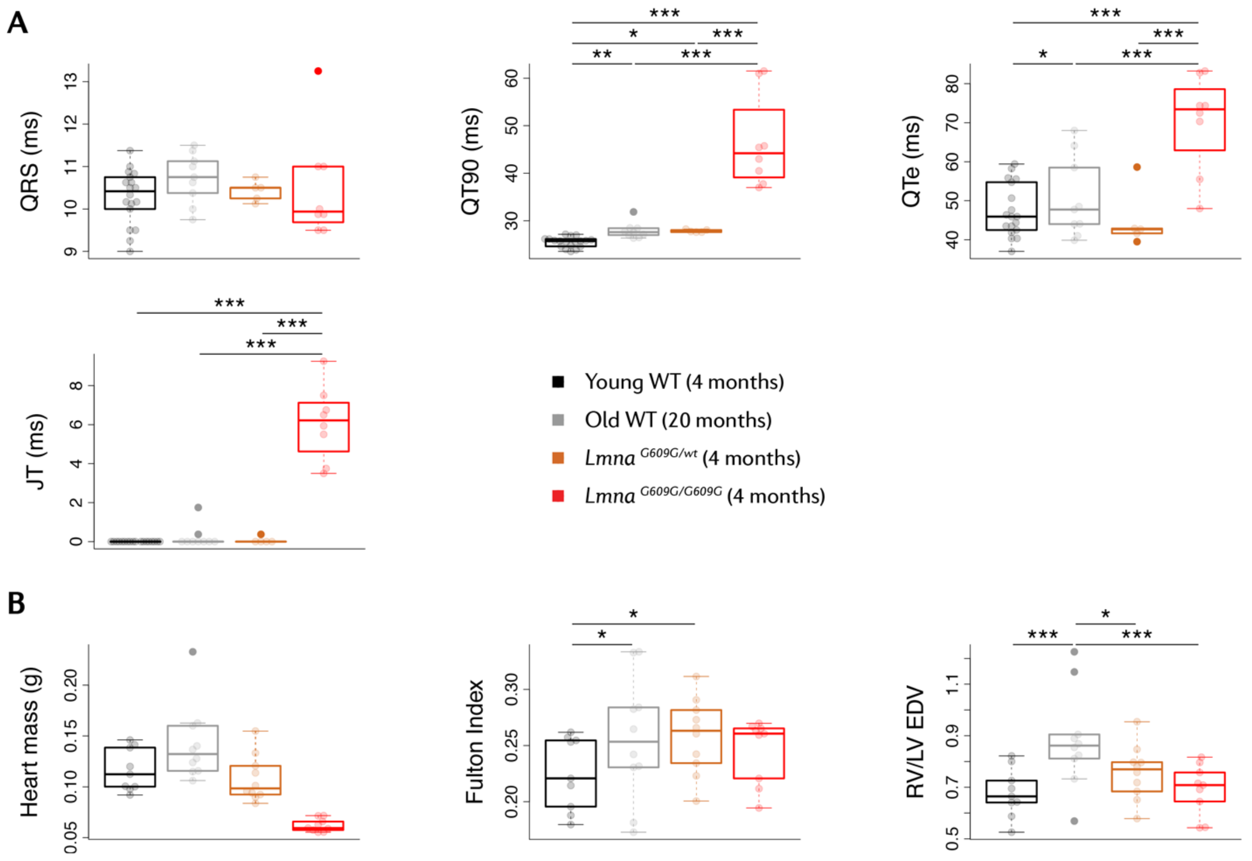

Supplementary Figure 1. Cardiac electrical and functional alterations in 4-month-old progeric *Lmna*$^{G609G/G609G}$ and *Lmna*$^{G609G/wt}$ mice and 4-month-old and 20-month-old WT mice. **A)** ECG parameters (n=8 *Lmna*$^{G609G/G609G}$, 5 *Lmna*$^{G609G/wt}$, 18 young WT and 9 Old WT mice). Measurements were performed at least 3 times within a month. **B)** Cardiac function parameters derived from MRI (n=10 mice per group). Body weight was considered as a covariate in the statistical analysis of heart mass. *, p<0.05; **, p<0.01; ***, p<0.001.

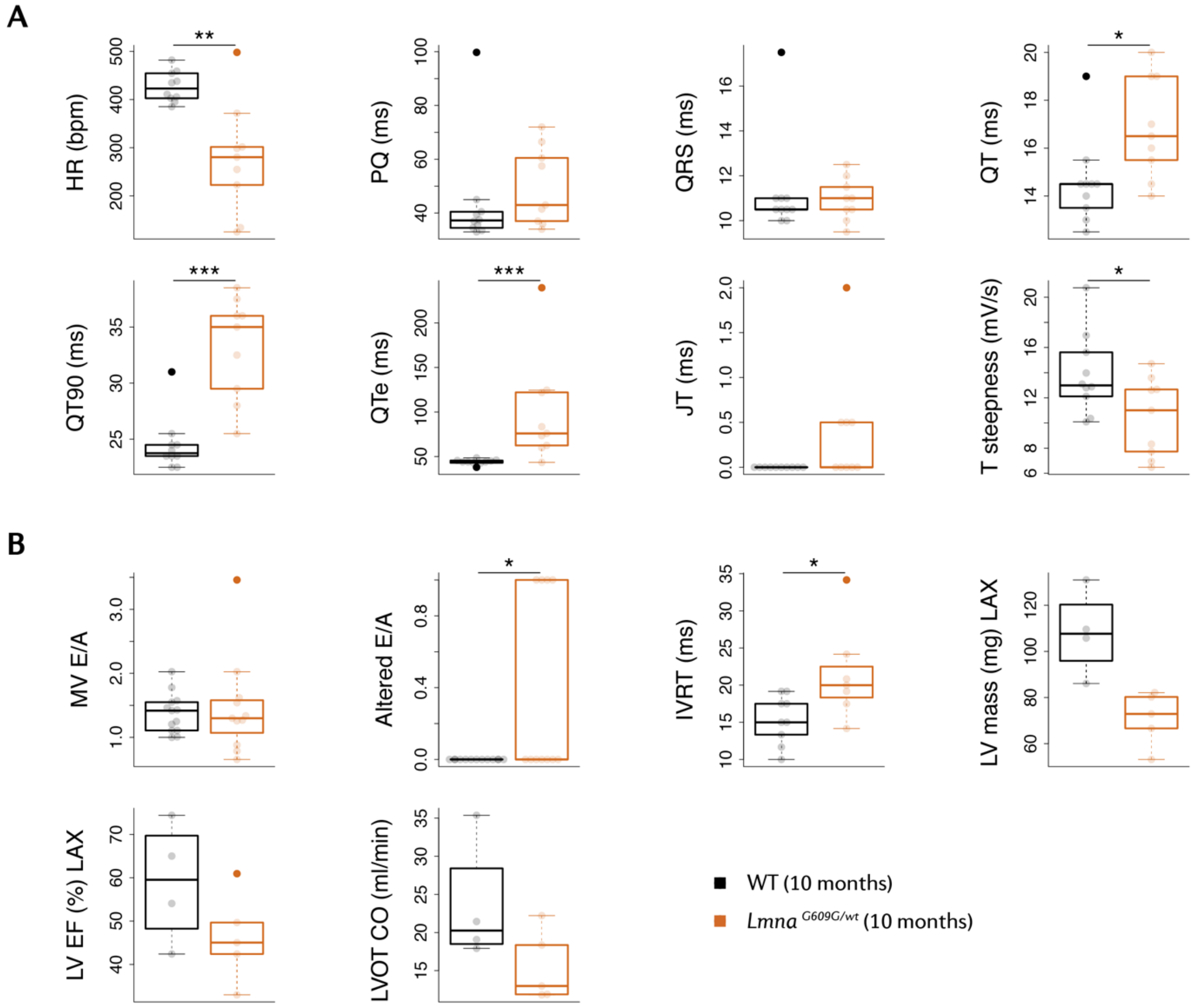

Supplementary Figure 2. Cardiac electrical and functional alterations in 10-month-old progeric *Lmna*$^{G609G/wt}$ and age-matched WT mice. A) ECG parameters (n=9-10 mice per group). **B)** Echocardiographic parameters (n=12-13 mice per group). Body weight was considered as a covariate in the statistical analysis of LV mass and CO. E/A was contemplated as altered when animals presented restrictive LV filling (E/A>3) or impaired relaxation (E/A < 1). LAX, long axis view; LVOT, LV outflow tract. *, p<0.05; **, p<0.01; ***, p<0.001.

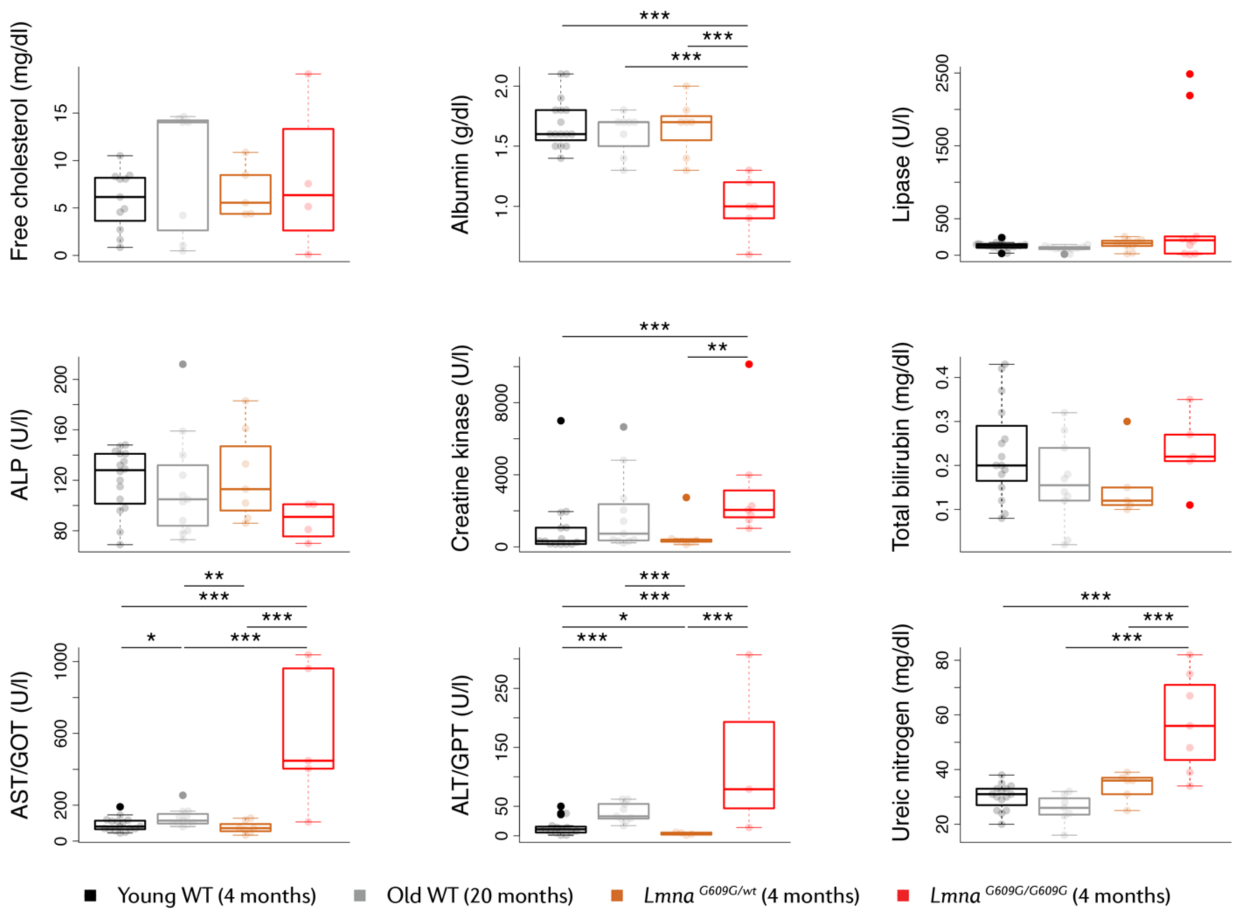

Supplementary Figure 3. Biochemical analysis of metabolism-related serum parameters in 4-month-old progeric *Lmna*$^{G609G/G609G}$ and *Lmna*$^{G609G/wt}$ mice and age-matched and 20-month-old WT animals (n=10-16 mice per group). ALP, alkaline phosphatase; AST, aspartate transaminase; GOT, oxaloacetic transaminase; ALT, alanine transaminase; GPT, glutamate pyruvate transaminase. *, p<0.05; **, p<0.01; ***, p<0.001.

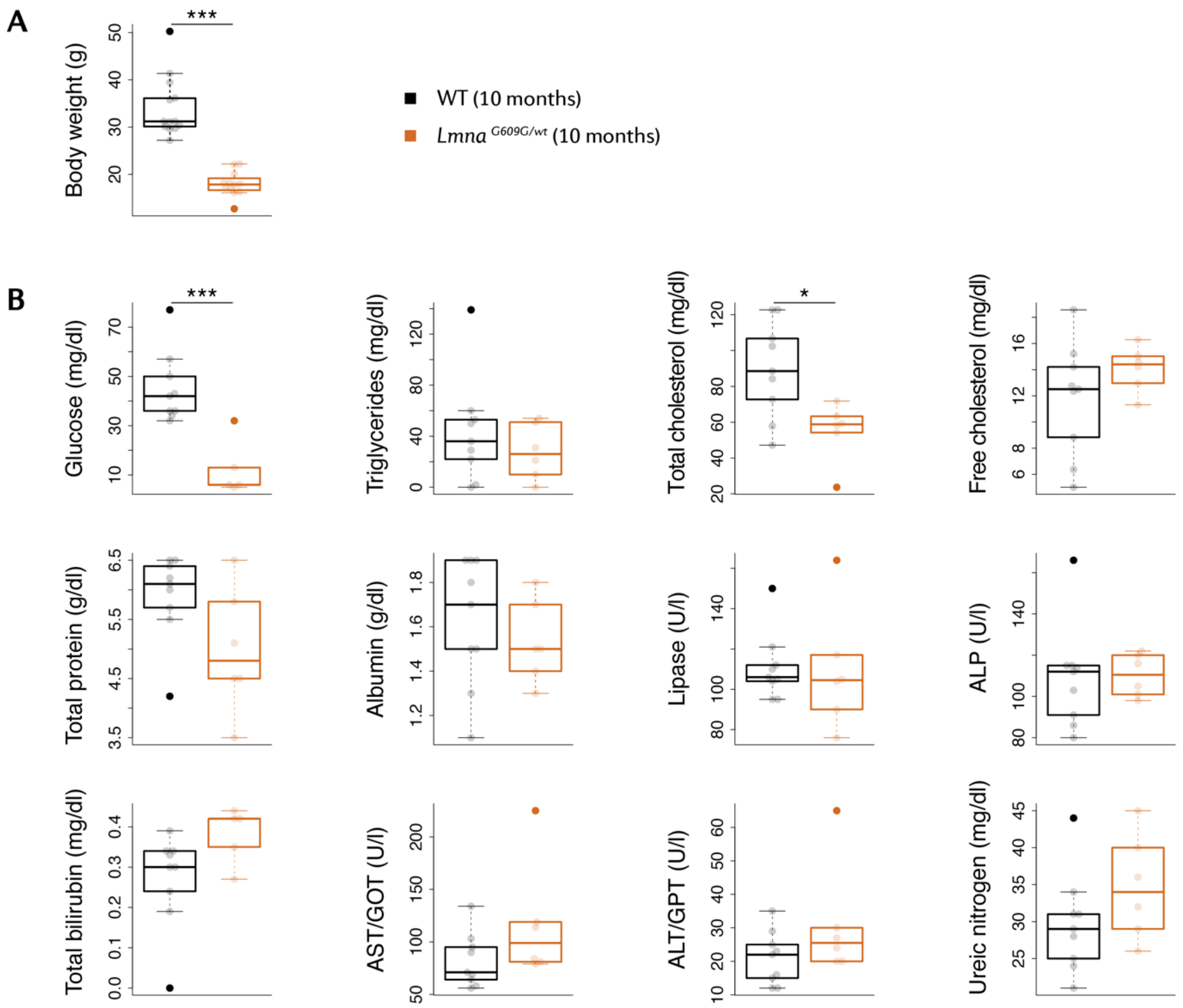

Supplementary Figure 4. Systemic metabolic alterations in 10-month-old progeric *Lmna*$^{G609G/wt}$ and age-matched WT mice. **A)** Body weight (n=12-13 mice per group). **B)** Biochemical analysis of metabolism-related serum parameters (n=6-9 mice per group). ALP, alkaline phosphatase; AST, aspartate transaminase; GOT, oxaloacetic transaminase; ALT, alanine transaminase; GPT, glutamate pyruvate transaminase. *, p<0.05; **, p<0.01; ***, p<0.001.

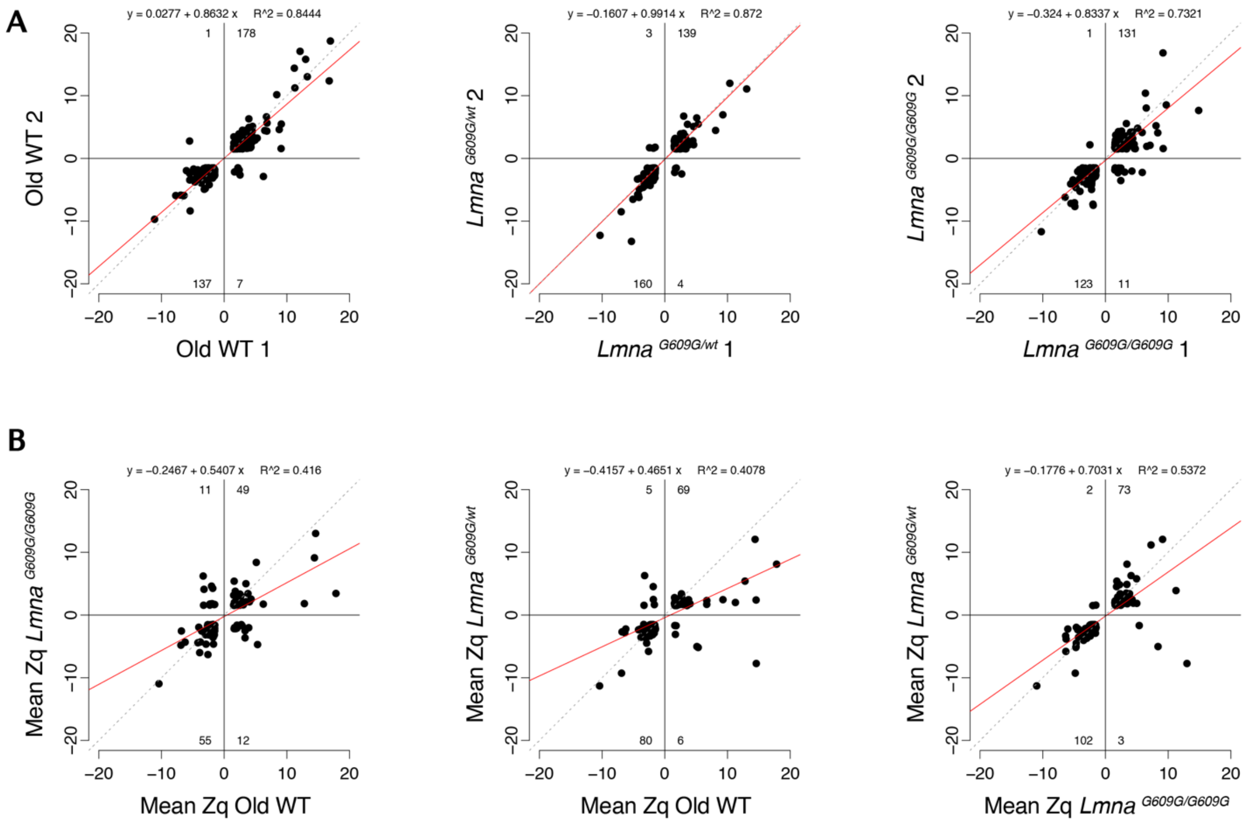

Supplementary Figure 5. Correlations between biological replicates **(A)** and between conditions **(B)** for proteins quantified with >1 peptide and |mean Zq|>1.5. Studies were carried out in hearts from 4-month-old progeric *Lmna*$^{G609G/G609G}$ and *Lmna*$^{G609G/wt}$ mice and from 4-month-old (Young) and 20-month-old (Old) WT mice.

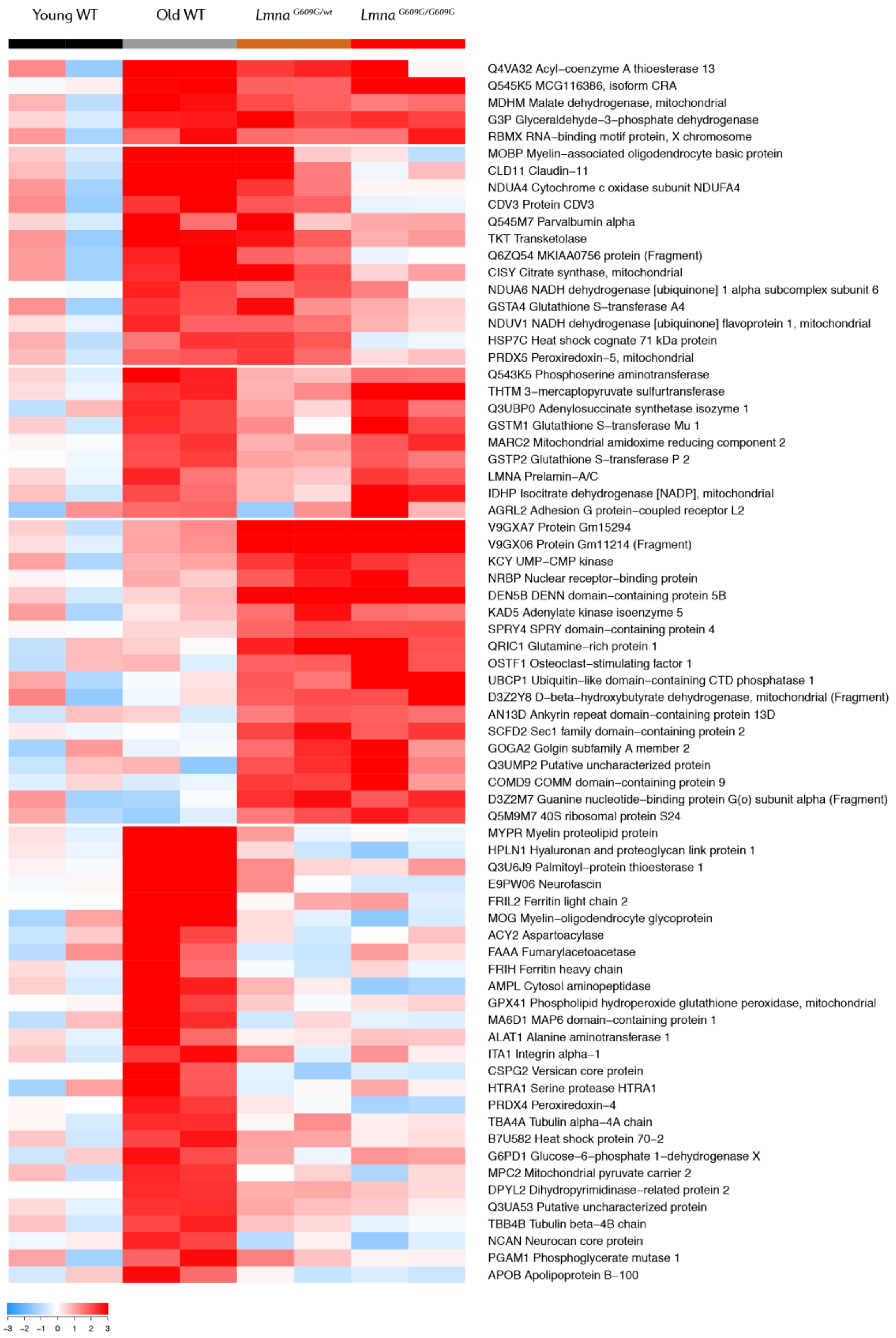
Young WT
Old WT
Lmna G609G/wt
Lmna G609G/G609G
Q4VA32 Acyl–coenzyme A thioesterase 13
Q545K5 MCG116386, isoform CRA
MDHM Malate dehydrogenase, mitochondrial
G3P Glyceraldehyde–3–phosphate dehydrogenase
RBMX RNA–binding motif protein, X chromosome
MOBP Myelin–associated oligodendrocyte basic protein
CLD11 Claudin–11
NDUA4 Cytochrome c oxidase subunit NDUFA4
CDV3 Protein CDV3
Q545M7 Parvalbumin alpha
TKT Transketolase
Q6ZQ54 MKIAA0756 protein (Fragment)
CISY Citrate synthase, mitochondrial
NDUA6 NADH dehydrogenase [ubiquinone] 1 alpha subcomplex subunit 6
GSTA4 Glutathione S–transferase A4
NDUV1 NADH dehydrogenase [ubiquinone] flavoprotein 1, mitochondrial
HSP7C Heat shock cognate 71 kDa protein
PRDX5 Peroxiredoxin–5, mitochondrial
Q543K5 Phosphoserine aminotransferase
THTM 3–mercaptopyruvate sulfurtransferase
Q3UBP0 Adenylosuccinate synthetase isozyme 1
GSTM1 Glutathione S–transferase Mu 1
MARC2 Mitochondrial amidoxime reducing component 2
GSTP2 Glutathione S–transferase P 2
LMNA Prelamin–A/C
IDHP Isocitrate dehydrogenase [NADP], mitochondrial
AGRL2 Adhesion G protein–coupled receptor L2
V9GXA7 Protein Gm15294
V9GX06 Protein Gm11214 (Fragment)
KCY UMP–CMP kinase
NRBP Nuclear receptor–binding protein
DEN5B DENN domain–containing protein 5B
KAD5 Adenylate kinase isoenzyme 5
SPRY4 SPRY domain–containing protein 4
QRIC1 Glutamine–rich protein 1
OSTF1 Osteoclast–stimulating factor 1
UBCP1 Ubiquitin–like domain–containing CTD phosphatase 1
D3Z2Y8 D–beta–hydroxybutyrate dehydrogenase, mitochondrial (Fragment)
AN13D Ankyrin repeat domain–containing protein 13D
SCFD2 Sec1 family domain–containing protein 2
GOGA2 Golgin subfamily A member 2
Q3UMP2 Putative uncharacterized protein
COMD9 COMM domain–containing protein 9
D3Z2M7 Guanine nucleotide–binding protein G(o) subunit alpha (Fragment)
Q5M9M7 40S ribosomal protein S24
MYPR Myelin proteolipid protein
HPLN1 Hyaluronan and proteoglycan link protein 1
Q3U6J9 Palmitoyl–protein thioesterase 1
E9PW06 Neurofascin
FRIL2 Ferritin light chain 2
MOG Myelin–oligodendrocyte glycoprotein
ACY2 Aspartoacylase
FAAA Fumarylacetoacetase
FRIH Ferritin heavy chain
AMPL Cytosol aminopeptidase
GPX41 Phospholipid hydroperoxide glutathione peroxidase, mitochondrial
MA6D1 MAP6 domain–containing protein 1
ALAT1 Alanine aminotransferase 1
ITA1 Integrin alpha–1
CSPG2 Versican core protein
HTRA1 Serine protease HTRA1
PRDX4 Peroxiredoxin–4
TBA4A Tubulin alpha–4A chain
B7U582 Heat shock protein 70–2
G6PD1 Glucose–6–phosphate 1–dehydrogenase X
MPC2 Mitochondrial pyruvate carrier 2
DPYL2 Dihydropyrimidinase–related protein 2
Q3UA53 Putative uncharacterized protein
TBB4B Tubulin beta–4B chain
NCAN Neurocan core protein
PGAM1 Phosphoglycerate mutase 1
APOB Apolipoprotein B–100
–3 –2 –1 0 1 2 3

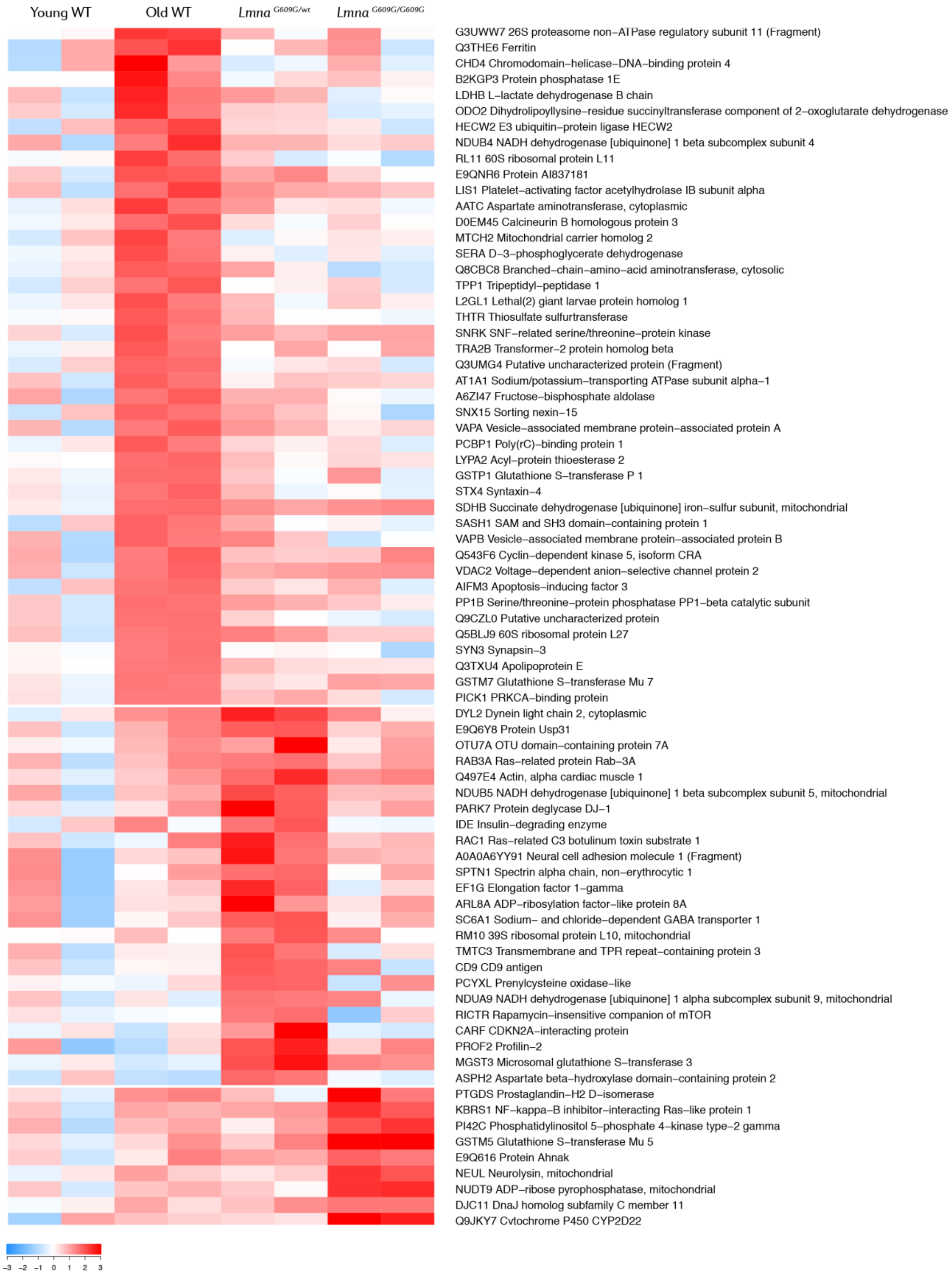
Young WT
Old WT
Lmna G609G/wt
Lmna G609G/G609G
G3UWW7 26S proteasome non–ATPase regulatory subunit 11 (Fragment)
Q3THE6 Ferritin
CHD4 Chromodomain–helicase–DNA–binding protein 4
B2KGP3 Protein phosphatase 1E
LDHB L–lactate dehydrogenase B chain
ODO2 Dihydrolipoyllysine–residue succinyltransferase component of 2–oxoglutarate dehydrogenase
HECW2 E3 ubiquitin–protein ligase HECW2
NDUB4 NADH dehydrogenase [ubiquinone] 1 beta subcomplex subunit 4
RL11 60S ribosomal protein L11
E9QNR6 Protein AI837181
LIS1 Platelet–activating factor acetylhydrolase IB subunit alpha
AATC Aspartate aminotransferase, cytoplasmic
D0EM45 Calcineurin B homologous protein 3
MTCH2 Mitochondrial carrier homolog 2
SERA D–3–phosphoglycerate dehydrogenase
Q8CBC8 Branched–chain–amino–acid aminotransferase, cytosolic
TPP1 Tripeptidyl–peptidase 1
L2GL1 Lethal(2) giant larvae protein homolog 1
THTR Thiosulfate sulfurtransferase
SNRK SNF–related serine/threonine–protein kinase
TRA2B Transformer–2 protein homolog beta
Q3UMG4 Putative uncharacterized protein (Fragment)
AT1A1 Sodium/potassium–transporting ATPase subunit alpha–1
A6ZI47 Fructose–bisphosphate aldolase
SNX15 Sorting nexin–15
VAPA Vesicle–associated membrane protein–associated protein A
PCBP1 Poly(rC)–binding protein 1
LYPA2 Acyl–protein thioesterase 2
GSTP1 Glutathione S–transferase P 1
STX4 Syntaxin–4
SDHB Succinate dehydrogenase [ubiquinone] iron–sulfur subunit, mitochondrial
SASH1 SAM and SH3 domain–containing protein 1
VAPB Vesicle–associated membrane protein–associated protein B
Q543F6 Cyclin–dependent kinase 5, isoform CRA
VDAC2 Voltage–dependent anion–selective channel protein 2
AIFM3 Apoptosis–inducing factor 3
PP1B Serine/threonine–protein phosphatase PP1–beta catalytic subunit
Q9CZL0 Putative uncharacterized protein
Q5BLJ9 60S ribosomal protein L27
SYN3 Synapsin–3
Q3TXU4 Apolipoprotein E
GSTM7 Glutathione S–transferase Mu 7
PICK1 PRKCA–binding protein
DYL2 Dynein light chain 2, cytoplasmic
E9Q6Y8 Protein Usp31
OTU7A OTU domain–containing protein 7A
RAB3A Ras–related protein Rab–3A
Q497E4 Actin, alpha cardiac muscle 1
NDUB5 NADH dehydrogenase [ubiquinone] 1 beta subcomplex subunit 5, mitochondrial
PARK7 Protein deglycase DJ–1
IDE Insulin–degrading enzyme
RAC1 Ras–related C3 botulinum toxin substrate 1
A0A0A6YY91 Neural cell adhesion molecule 1 (Fragment)
SPTN1 Spectrin alpha chain, non–erythrocytic 1
EF1G Elongation factor 1–gamma
ARL8A ADP–ribosylation factor–like protein 8A
SC6A1 Sodium– and chloride–dependent GABA transporter 1
RM10 39S ribosomal protein L10, mitochondrial
TMTC3 Transmembrane and TPR repeat–containing protein 3
CD9 CD9 antigen
PCYXL Prenylcysteine oxidase–like
NDUA9 NADH dehydrogenase [ubiquinone] 1 alpha subcomplex subunit 9, mitochondrial
RICTR Rapamycin–insensitive companion of mTOR
CARF CDKN2A–interacting protein
PROF2 Profilin–2
MGST3 Microsomal glutathione S–transferase 3
ASPH2 Aspartate beta–hydroxylase domain–containing protein 2
PTGDS Prostaglandin–H2 D–isomerase
KBRS1 NF–kappa–B inhibitor–interacting Ras–like protein 1
PI42C Phosphatidylinositol 5–phosphate 4–kinase type–2 gamma
GSTM5 Glutathione S–transferase Mu 5
E9Q616 Protein Ahnak
NEUL Neurolysin, mitochondrial
NUDT9 ADP–ribose pyrophosphatase, mitochondrial
DJC11 DnaJ homolog subfamily C member 11
Q9JKY7 Cytochrome P450 CYP2D22
–3 –2 –1 0 1 2 3

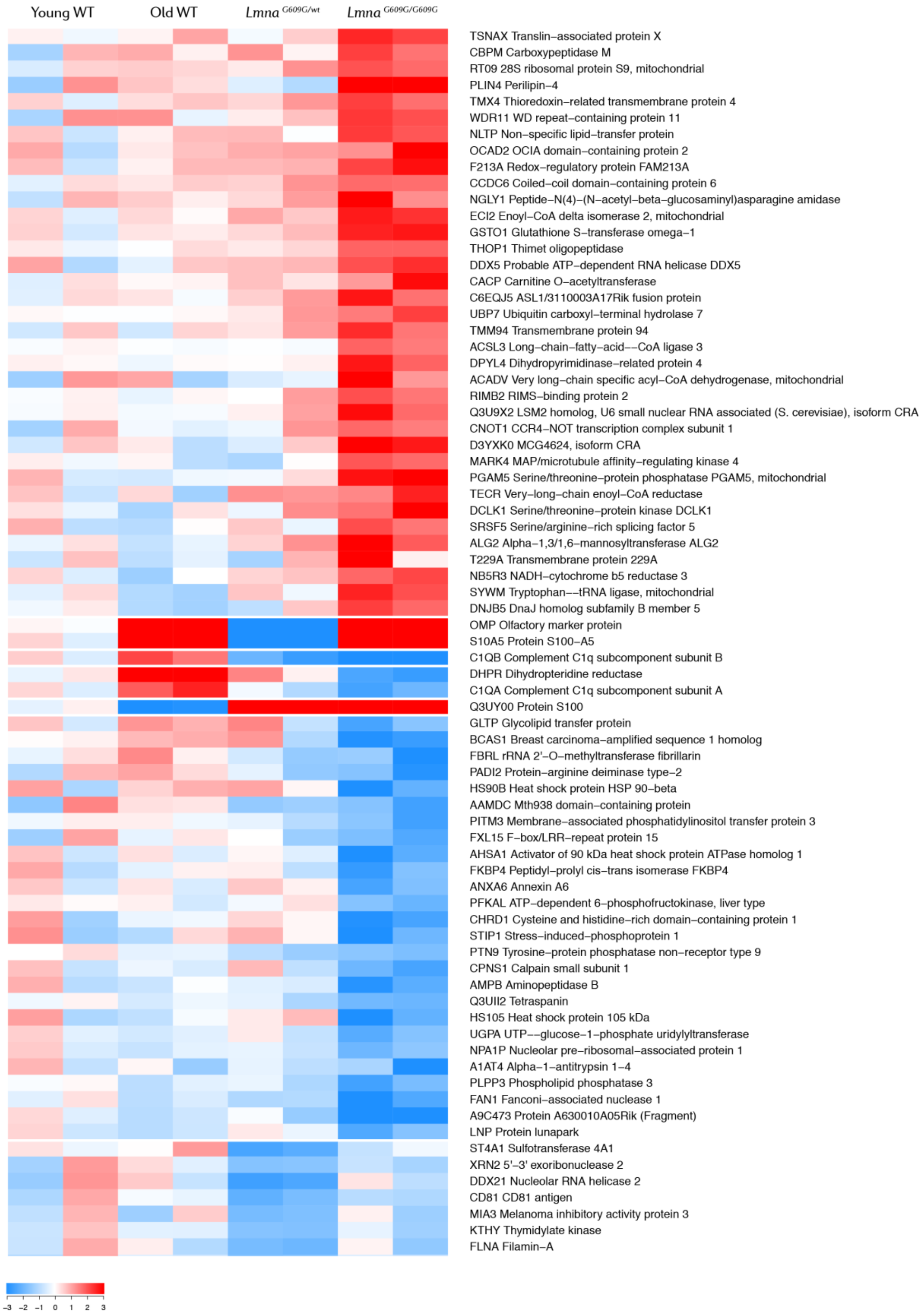
Young WT
Old WT
Lmna G609G/wt
Lmna G609G/G609G
TSNAX Translin–associated protein X
CBPM Carboxypeptidase M
RT09 28S ribosomal protein S9, mitochondrial
PLIN4 Perilipin–4
TMX4 Thioredoxin–related transmembrane protein 4
WDR11 WD repeat–containing protein 11
NLTP Non–specific lipid–transfer protein
OCAD2 OCIA domain–containing protein 2
F213A Redox–regulatory protein FAM213A
CCDC6 Coiled–coil domain–containing protein 6
NGLY1 Peptide–N(4)–(N–acetyl–beta–glucosaminyl)asparagine amidase
ECI2 Enoyl–CoA delta isomerase 2, mitochondrial
GSTO1 Glutathione S–transferase omega–1
THOP1 Thimet oligopeptidase
DDX5 Probable ATP–dependent RNA helicase DDX5
CACP Carnitine O–acetyltransferase
C6EQJ5 ASL1/3110003A17Rik fusion protein
UBP7 Ubiquitin carboxyl–terminal hydrolase 7
TMM94 Transmembrane protein 94
ACSL3 Long–chain–fatty–acid––CoA ligase 3
DPYL4 Dihydropyrimidinase–related protein 4
ACADV Very long–chain specific acyl–CoA dehydrogenase, mitochondrial
RIMB2 RIMS–binding protein 2
Q3U9X2 LSM2 homolog, U6 small nuclear RNA associated (S. cerevisiae), isoform CRA
CNOT1 CCR4–NOT transcription complex subunit 1
D3YXK0 MCG4624, isoform CRA
MARK4 MAP/microtubule affinity–regulating kinase 4
PGAM5 Serine/threonine–protein phosphatase PGAM5, mitochondrial
TECR Very–long–chain enoyl–CoA reductase
DCLK1 Serine/threonine–protein kinase DCLK1
SRSF5 Serine/arginine–rich splicing factor 5
ALG2 Alpha–1,3/1,6–mannosyltransferase ALG2
T229A Transmembrane protein 229A
NB5R3 NADH–cytochrome b5 reductase 3
SYWM Tryptophan––tRNA ligase, mitochondrial
DNJB5 DnaJ homolog subfamily B member 5
OMP Olfactory marker protein
S10A5 Protein S100–A5
C1QB Complement C1q subcomponent subunit B
DHPR Dihydropteridine reductase
C1QA Complement C1q subcomponent subunit A
Q3UY00 Protein S100
GLTP Glycolipid transfer protein
BCAS1 Breast carcinoma–amplified sequence 1 homolog
FBRL rRNA 2'–O–methyltransferase fibrillarin
PADI2 Protein–arginine deiminase type–2
HS90B Heat shock protein HSP 90–beta
AAMDC Mth938 domain–containing protein
PITM3 Membrane–associated phosphatidylinositol transfer protein 3
FXL15 F–box/LRR–repeat protein 15
AHSA1 Activator of 90 kDa heat shock protein ATPase homolog 1
FKBP4 Peptidyl–prolyl cis–trans isomerase FKBP4
ANXA6 Annexin A6
PFKAL ATP–dependent 6–phosphofructokinase, liver type
CHRD1 Cysteine and histidine–rich domain–containing protein 1
STIP1 Stress–induced–phosphoprotein 1
PTN9 Tyrosine–protein phosphatase non–receptor type 9
CPNS1 Calpain small subunit 1
AMPB Aminopeptidase B
Q3UII2 Tetraspanin
HS105 Heat shock protein 105 kDa
UGPA UTP––glucose–1–phosphate uridylyltransferase
NPA1P Nucleolar pre–ribosomal–associated protein 1
A1AT4 Alpha–1–antitrypsin 1–4
PLPP3 Phospholipid phosphatase 3
FAN1 Fanconi–associated nuclease 1
A9C473 Protein A630010A05Rik (Fragment)
LNP Protein lunapark
ST4A1 Sulfotransferase 4A1
XRN2 5'–3' exoribonuclease 2
DDX21 Nucleolar RNA helicase 2
CD81 CD81 antigen
MIA3 Melanoma inhibitory activity protein 3
KTHY Thymidylate kinase
FLNA Filamin–A
–3 –2 –1 0 1 2 3

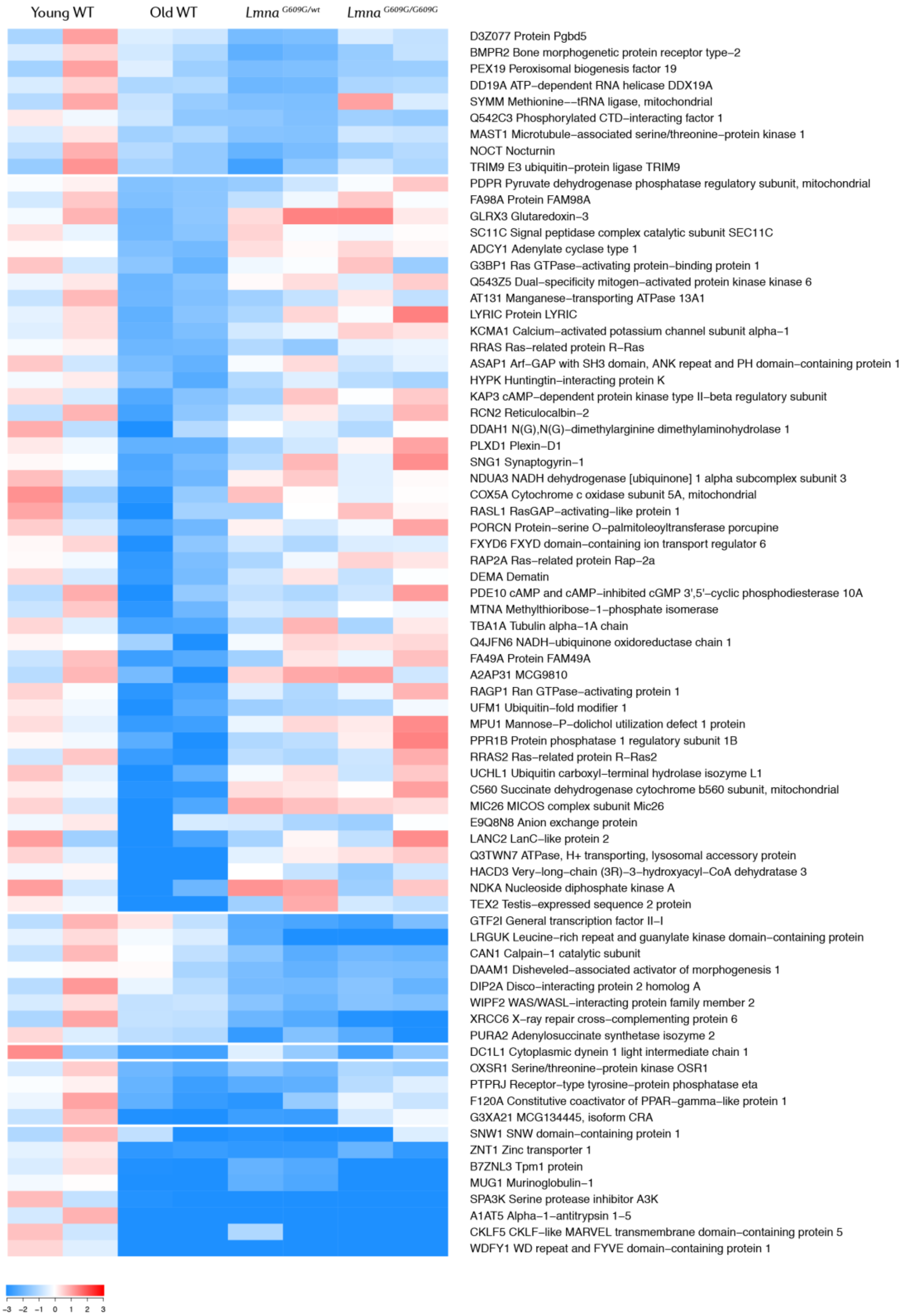

Supplementary Figure 6. Relative protein abundance changes in the aging mouse heart. Heatmap of curated protein candidates with altered expression in 4-month-old progeric *Lmna*$^{G609G/G609G}$ and *Lmna*$^{G609G/wt}$ mice and in 4-month-old (Young) and 20-month-old (Old) WT mice. Zq indicates protein abundance compared with young WT mice. Selected proteins were quantified with >1 unique peptide and had |Zq|>1.5 in both replicates or |mean Zq|>1.5. Proteins with inconsistent changes between replicates were excluded. Color intensity is maximal when |Zq|≥3.

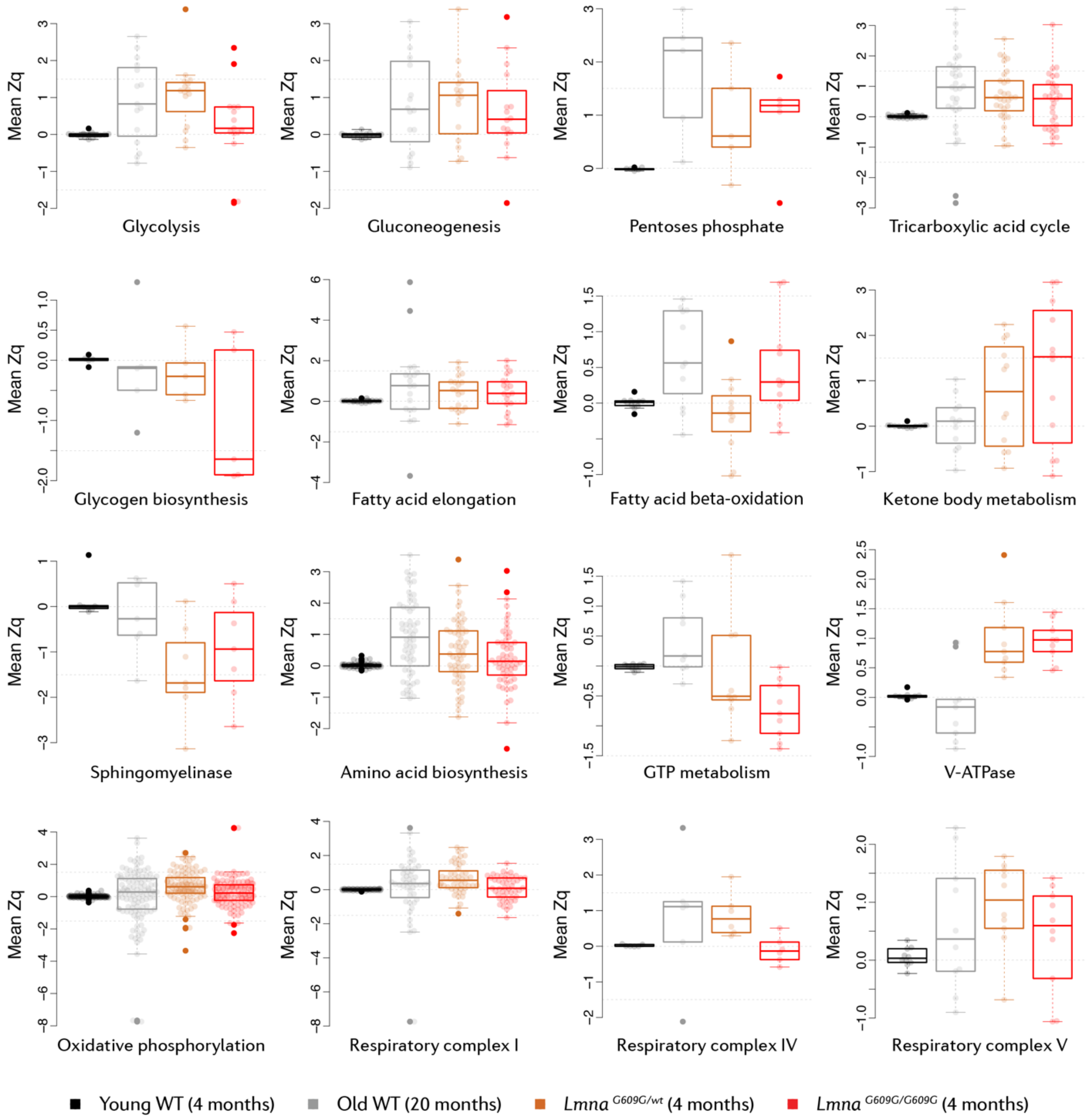

Supplementary Figure 7. Main energy metabolism-related pathways altered in the aging mouse heart proteome. Representative pathways with significant changes were extracted from the systems biology analysis. Each dot represents a protein in the pathway. Zq indicates protein abundance compared with young WT mice.

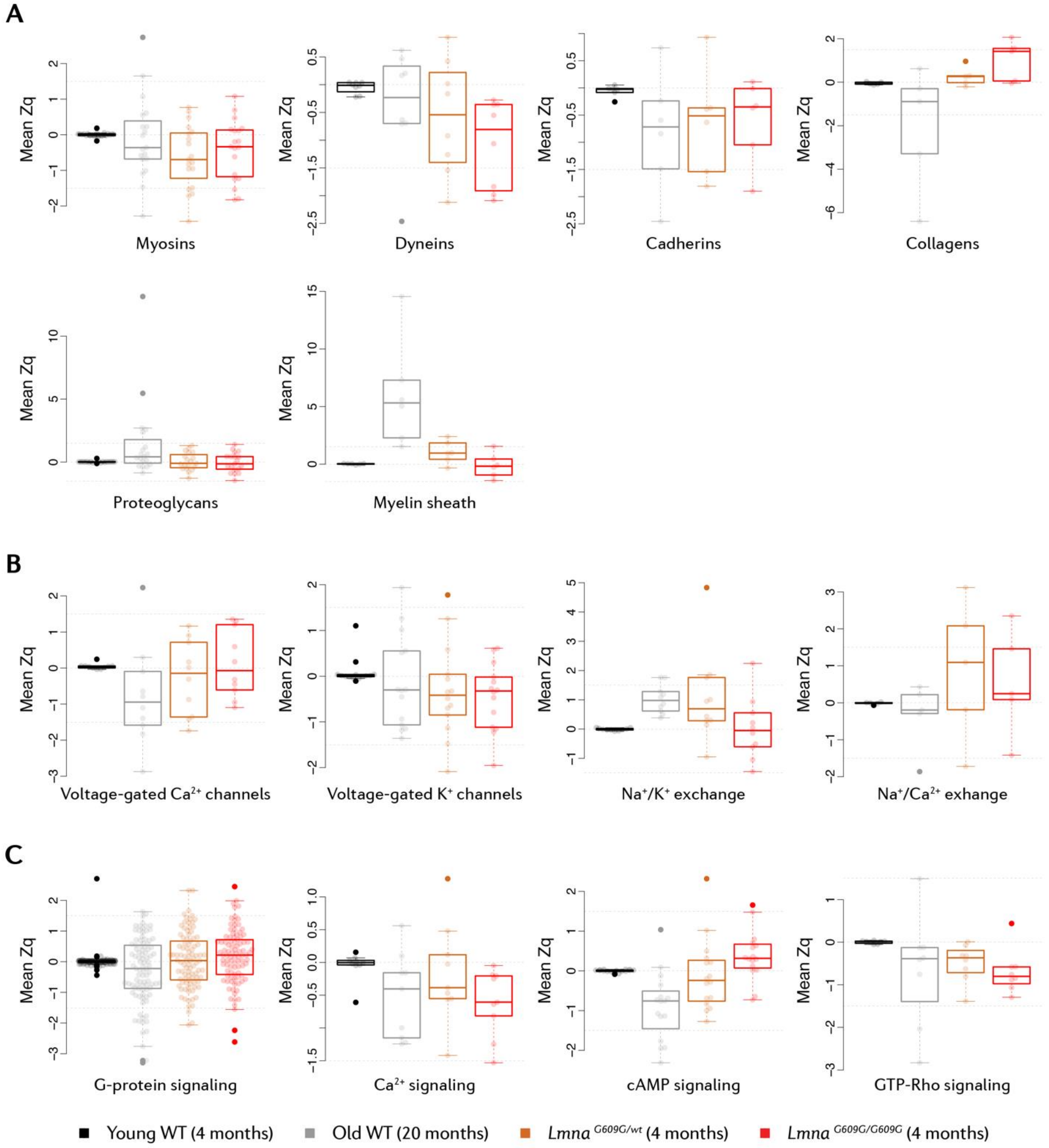

Supplementary Figure 8. Main cardiac contraction-related pathways altered in the aging mouse heart proteome. Representative pathways with significant changes were extracted from the systems biology analysis. **A)** Structural and motor proteins. **B)** Ion channels. **C)** Signaling pathways. Each dot represents a protein in the pathway. Zq indicates protein abundance compared with young WT mice.

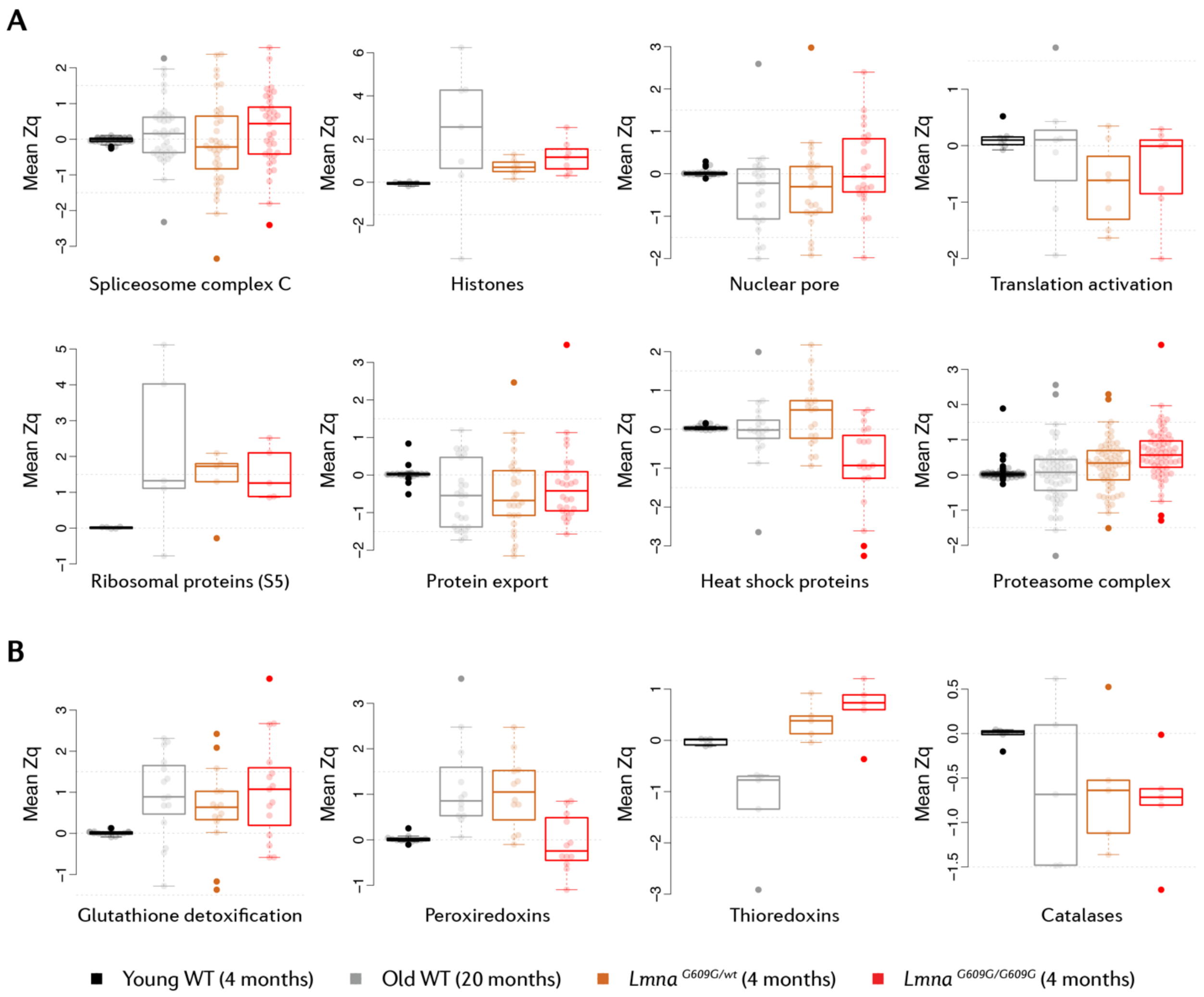

Supplementary Figure 9. Main homeostasis-related pathways altered in the aging mouse heart proteome. Representative pathways with significant changes were extracted from the systems biology analysis. **A)** Gene expression & proteostasis. **B)** Antioxidant response. Each dot represents a protein in the pathway. Zq indicates protein abundance compared with young WT mice.

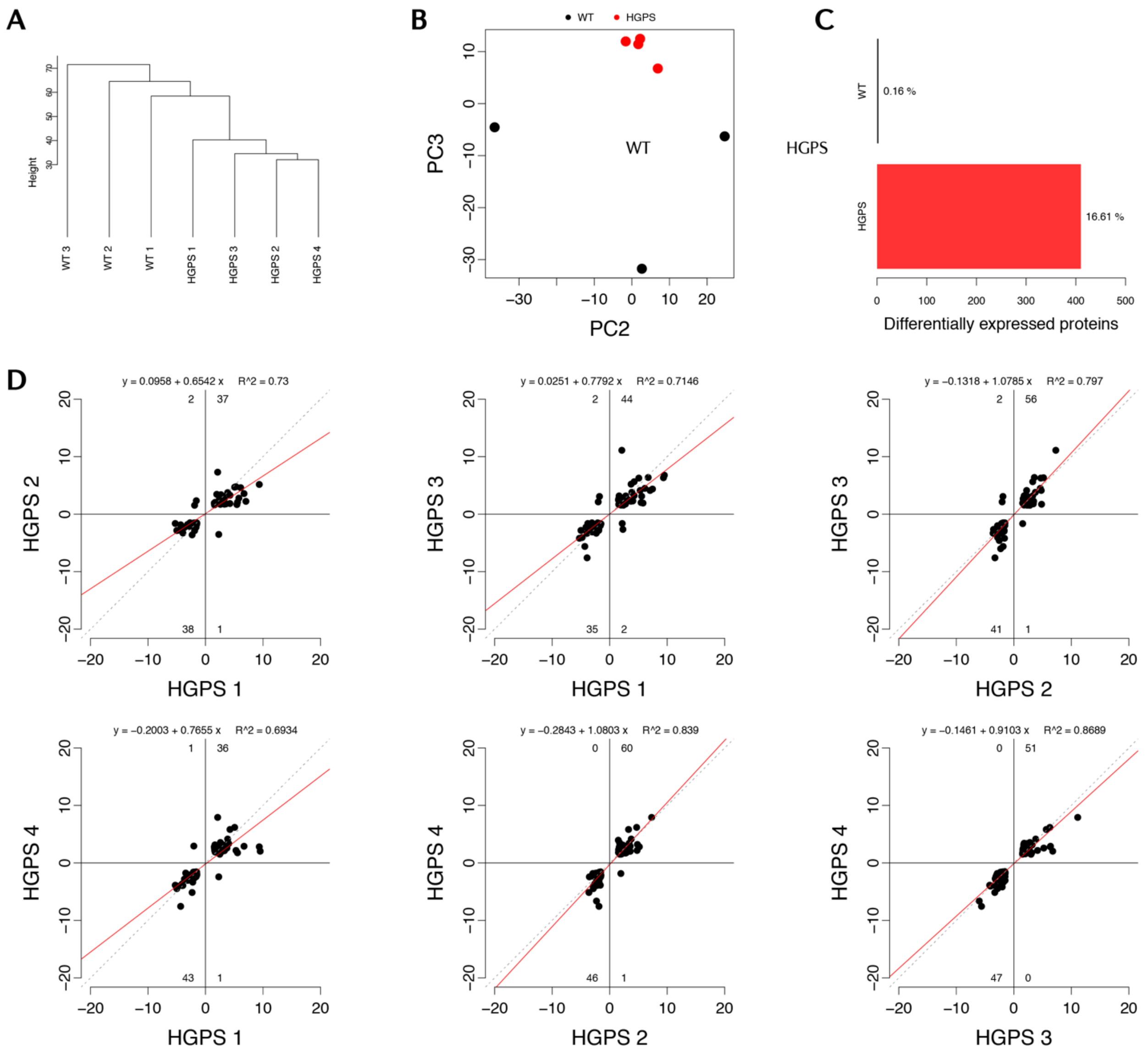

Supplementary Figure 10. High-throughput proteomics in the progeric pig heart. **A)** Hierarchical clustering and **B)** PCA of the samples (filtering for proteins with >1 unique peptide). **C)** Bar plots representing the number of differentially expressed proteins in each condition. **D)** Correlations between biological replicates for proteins with altered expression. A protein was considered significantly changed when it was quantified with >1 unique peptide and the mean Zq between replicates was >1.5.

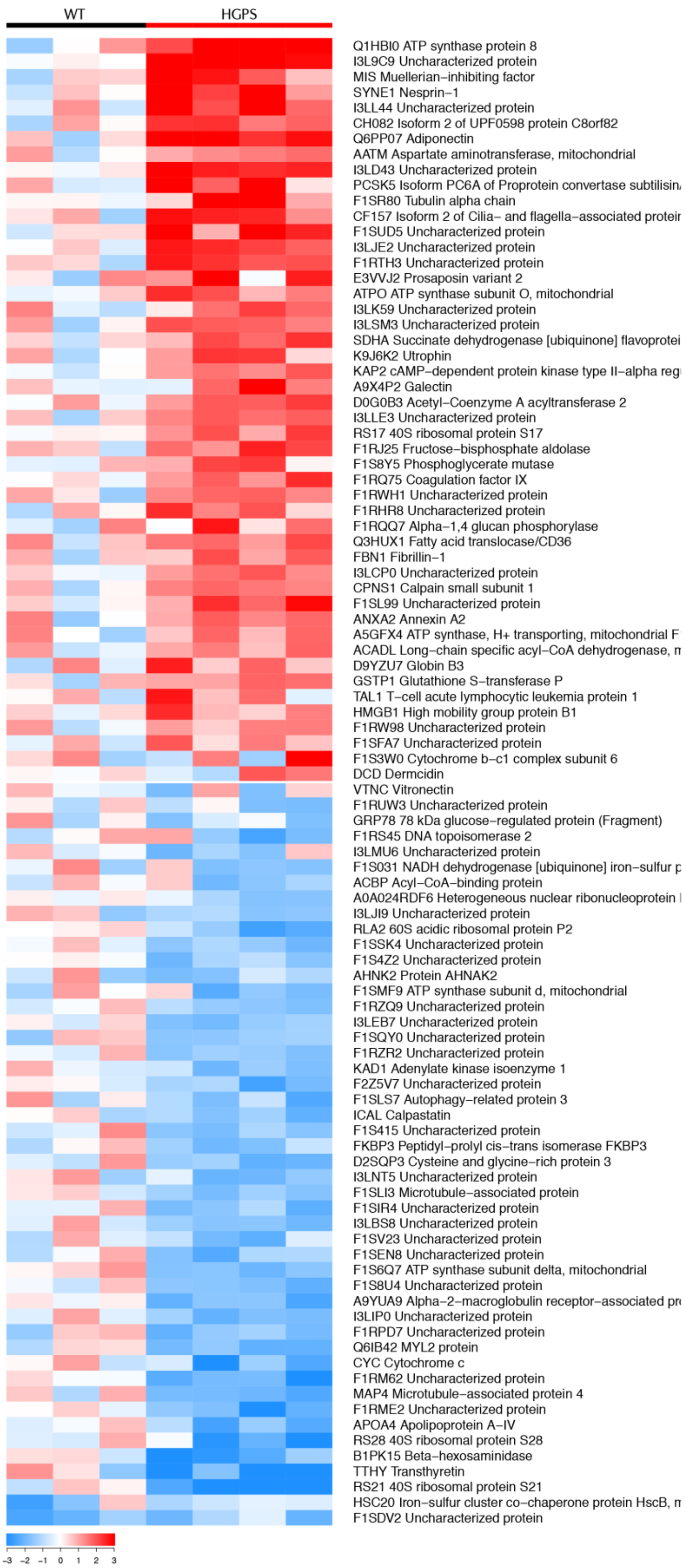

Supplementary Figure 11. Relative protein abundance changes in the progeric pig heart. Heatmap of curated protein candidates with altered expression in the progeric pig model. Zq indicates protein abundance compared with WT pigs. Selected proteins were quantified with >1 unique peptide and had |Zq|>1.5 in at least two replicates or |mean Zq|>1.5. Proteins with inconsistent changes between replicates were excluded. Color intensity is maximal when |Zq|≥3.

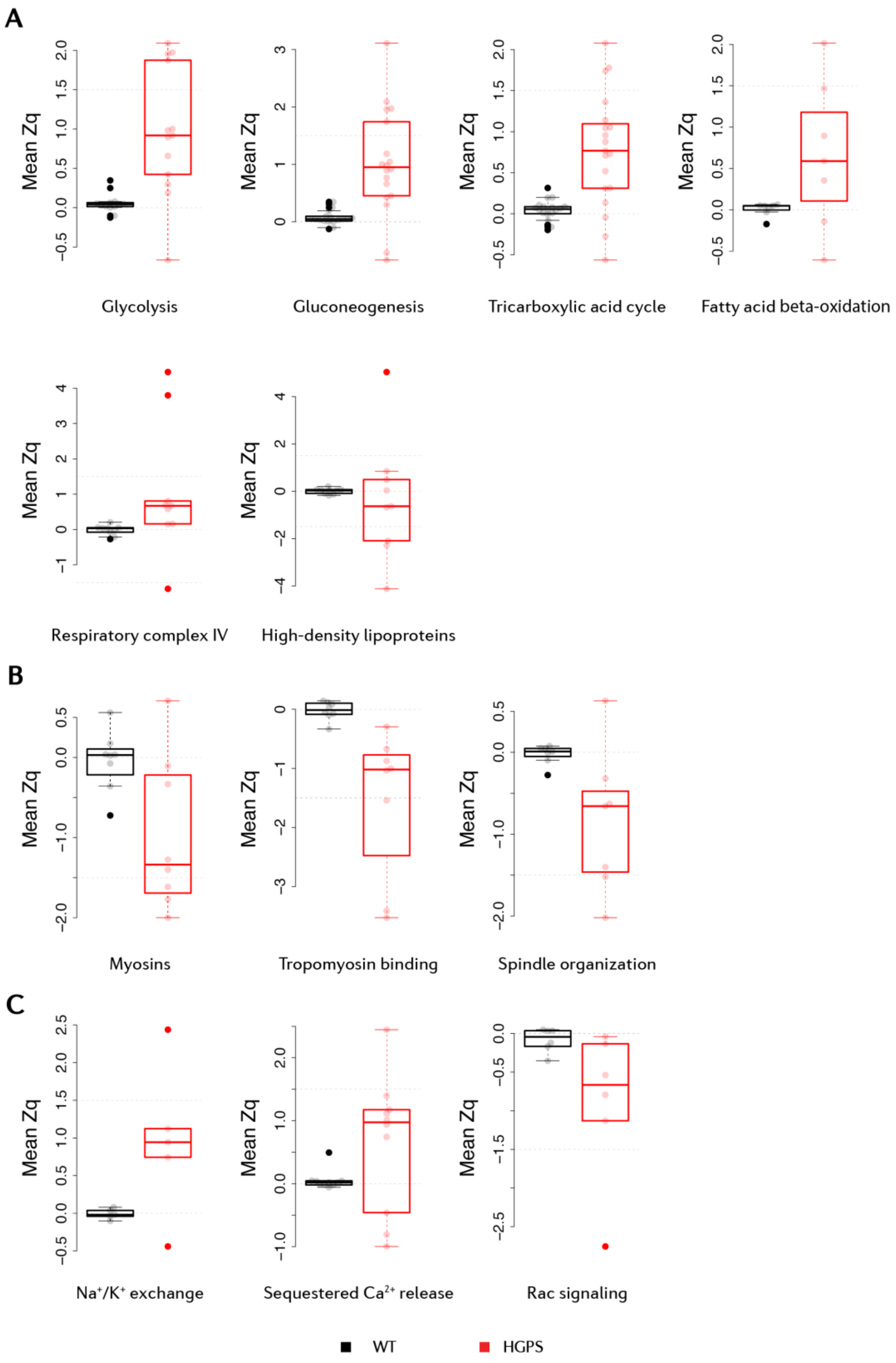

Supplementary Figure 12. Main pathways altered in the progeric pig heart proteome. Representative pathways with significant changes were extracted from the systems biology analysis. **A)** Energy metabolism-related pathways. **B)** Structural and motor proteins. C) Ion channels and signaling pathways. Each dot represents a protein in the pathway. Zq indicates protein abundance compared with WT pigs.

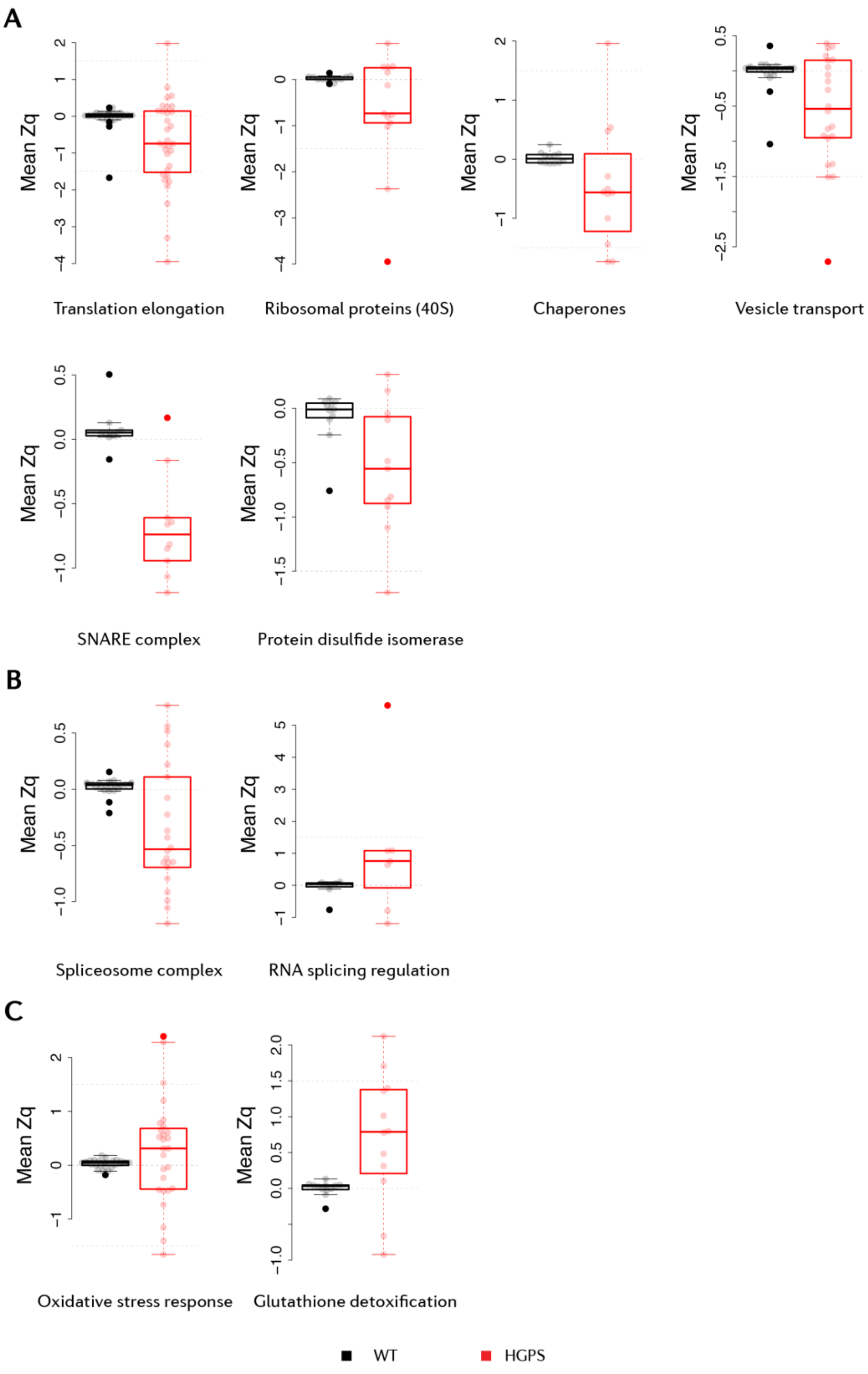

Supplementary Figure 13. Main pathways altered in the progeric pig heart proteome. Representative pathways with significant changes were extracted from the systems biology analysis. **A)** Proteostasis. **B)** Gene expression. **C)** Antioxidant response. Each dot represents a protein in the pathway. Zq indicates protein abundance compared with WT pigs.

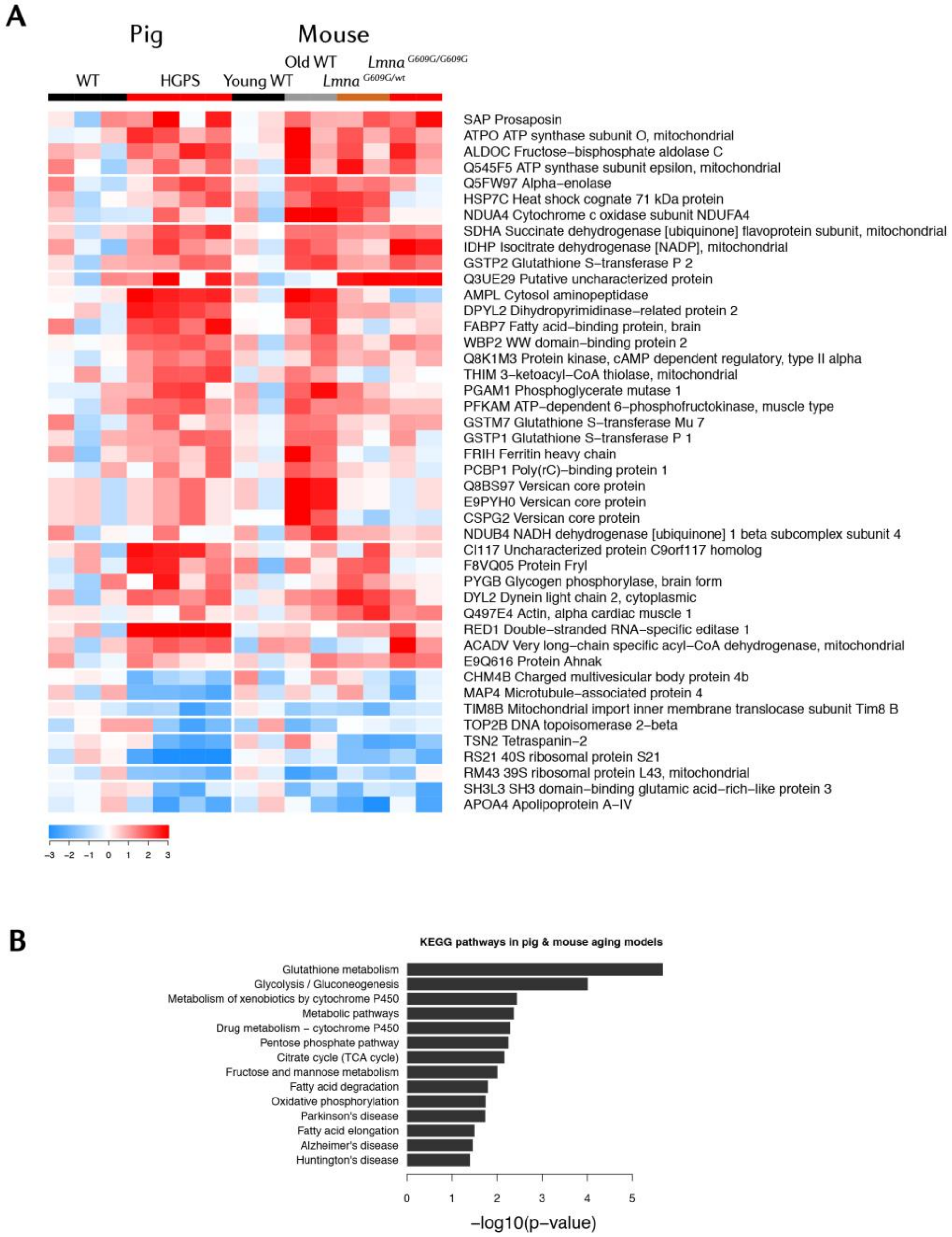

Supplementary Figure 14. A) Heatmap of curated protein candidates with altered expression in mouse and pig models of premature and/or normal aging. Selected proteins were quantified with >1 unique peptide and had |Zq|>1.5 in more than one replicate or |mean Zq|>1.5. Proteins with inconsistent changes between replicates were excluded. Color intensity is maximal when |Zq|≥3. **B)** Gene enrichment analysis of proteins with altered expression in premature and/or normal aging mouse and pig models.
